# Supplementary material for: Ergodicity-breaking reveals time optimal decision making in humans
Source: PLoS Comput Biol. 2021 Sep 9;17(9):e1009217. doi: 10.1371/journal.pcbi.1009217 (PMC8454984; doi:10.1371/journal.pcbi.1009217)
Supplement: S1 Text — S1 Fig in S1 Text. Growth rates for outcomes and gambles. A, on Day× (upper table) a growth factor is the factor by which current wealth is multiplied when a given stimulus is encountered in the passive session. The effect of each stimulus can thus be expressed as a multiplicative growth rate (in units of growth factor per trial). Computing the natural logarithm of the growth factor per trial gives a continuous growth rate (in units of % change per trial). On Day+ (lower table), growth increments are the additive amounts by which wealth changes, and thus the growth rate is an additive growth rate (in units of DKK per trial). B, each gamble is comprised of two different possible outcomes, here denoted in terms of pairs of stimuli. Each cell shows the time average growth rate associated with each gamble in the space of possible gambles. The cells with red text indicate the 16 gambles that were presented in the active sessions. For Day× (upper) the time average multiplicative growth rates of each gamble have units % change per trial. For Day+ (lower) the time average additive growth rates have the units of DKK per trial. S2 Fig in S1 Text. Model comparisons and analysis of effects for choice proportions. A, the table of model probabilities, Bayes factors and error terms, for a repeated measures ANOVA on the choice proportions for discrepant trials. The meanings of each column are described in the text. B, the inclusion probabilities for all factors of interest across all models, along with the Bayes factors for their inclusion. S3 Fig in S1 Text. Descriptive statistics, priors & posteriors of hypothesis test, robustness tests and sequential analyses. A-D, effect of dynamics on changing risk aversion parameters. H-K, comparison of the deviation of each model predictions of risk aversion parameters to those observed. L-O, effect of deviating from time optimality on the time average growth rates of subjects’ choices, under additive dynamics. P-S, equivalent effect under m [file pcbi.1009217.s001.docx]

**S1 text: Supplementary Results and Modelling**

**
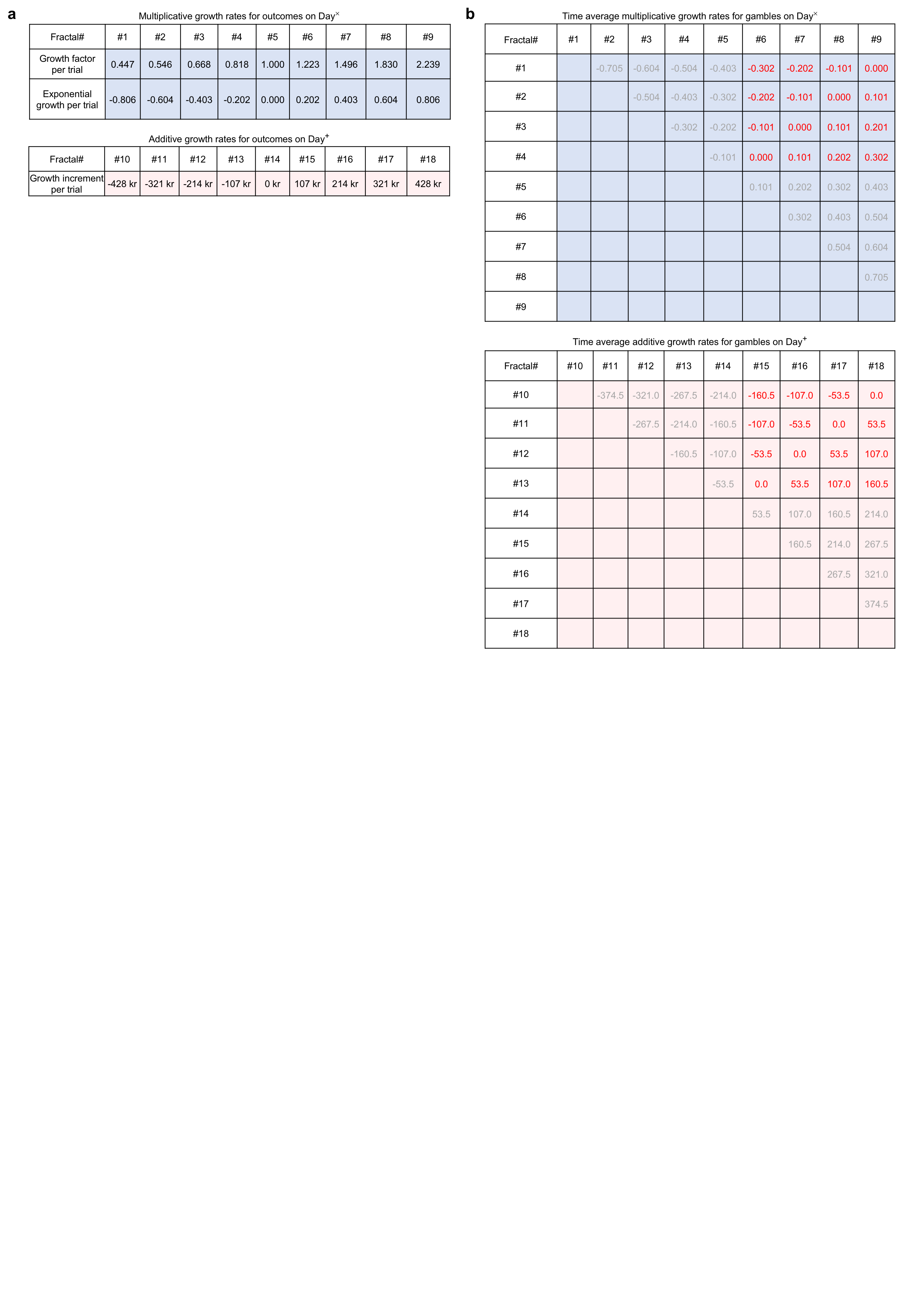
**

**S1 Fig** | **Growth rates for outcomes and gambles.** **A**, on Day^×^ (upper table) a growth factor is the factor by which current wealth is multiplied when a given stimulus is encountered in the passive session. The effect of each stimulus can thus be expressed as a multiplicative growth rate (in units of *growth factor per trial*). Computing the natural logarithm of the growth factor per trial gives a continuous growth rate (in units of *% change per trial*). On Day^+^ (lower table), growth increments are the additive amounts by which wealth changes, and thus the growth rate is an additive growth rate (in units of *DKK per trial*). **B,** each gamble is comprised of two different possible outcomes, here denoted in terms of pairs of stimuli. Each cell shows the time average growth rate associated with each gamble in the space of possible gambles. The cells with red text indicate the 16 gambles that were presented in the active sessions. For Day^×^ (upper) the time average multiplicative growth rates of each gamble have units *% change per trial*. For Day^+^ (lower) the time average additive growth rates have the units of*DKK per trial*.

**Choice proportion analysis Day^+^.** In the following H0 denotes the null hypothesis, H- to denotes the alternate hypothesis specifying values less than a reference value, and H+ to denote the equivalent for values above a reference value. Bayes factors obeys the same notation: BF_-0_ denotes a Bayes factor for H- over H0, BF_0-_  for H0 over H-, and so on. To assess choice proportions on Day^+^ we performed a one-sample Bayesian t-test in which we assign effect sizes a zero-centred Cauchy prior with scale 0.707 ($\frac{1}{\sqrt{2}}$). The fat-tailed Cauchy distribution is used because it fulfils particular criteria^1,2^. Of interest is the posterior distribution for the underlying choice proportion CP_log_. The resulting posterior distribution, which is concentrated near 0.5, with a central 95% credible interval of 0.395 to 0.591. The alternative hypothesis (H-) is relatively informative insofar as it states that CP_log_ is lower than 0.5, but that values of CP_log_ close to 0.5 are more likely than those values far below it (H- : 0 < CP_log_ < 0.5) as seen in Fig 2B which shows the one-sided prior and posterior distribution for the effect size of CP_log_ under the informative H-. Correspondingly, the null hypothesis (H0) states that agents will choose with respect to the linear utility less often than in favour of log utility, and thus predicts that the choice proportion in favour of log utility will be larger than 0.5 (H0: CP_log_ > 0.5). A one sample Bayesian t-test revealed a BF_0-_ of 3.678, which indicates the null hypothesis is nearly 4 times more likely than the alternative, which can be classed as moderate evidence. As shown in Fig 2B, compared to the prior distribution, the posterior distribution is more concentrated near an effect size of 0. For robustness checks, the effect of different prior widths (wide and ultrawide priors, scale factors 1 and $\surd2$, respectively) can be seen in Fig 2C and 2D, which show that they do not effectively change this interpretation. In conclusion, this indicates moderate evidence that under additive dynamics, choices in favour of linear utility were not more likely than those in favour of log utility.

**Choice proportion analysis Day^×^.** As above, to assess choice proportions on Day^×^ we performed a one-sample Bayesian t-test in which we assign effect sizes a zero-centred Cauchy prior, with scale 0.707. Of interest is the posterior distribution for the underlying choice proportion CP_log_. The resulting posterior distribution is concentrated near 0.7, with a central 95% credible interval for CP_log_ that ranges from 0.625 to 0.812. The alternative hypothesis is relatively informative and states that CP_log_ is higher than 0.5, but that values of CP_log_ close to 0.5 are more likely than values far above it (H+ : 1 > q > 0.5) as seen in Fig 2E which shows the one-sided prior and posterior distribution for the effect size of CP_log_ under the informative H+. The null hypothesis states that agents will not choose with respect to the log utility more often, and thus predicts that the choice proportion in favour of the log utility will be smaller than 0.5 (H0: CP_log_ < 0.5). A one sample Bayesian t-test revealed a Bayes Factor for the data being ~460 times more likely under H+ than under H0, which is classed as extreme evidence. As shown in Fig 2E, compared to the prior distribution, the posterior distribution is concentrated near an effect size of 1. Robustness checks and sequential analysis can be seen in Fig 2F and 2G, and do not effectively change this interpretation. In conclusion, this indicates extreme evidence that under multiplicative dynamics, choices in favour of log utility are more likely than those in favour of linear utility.

**Effect of dynamic on choice proportion.** To assess within subject changes in choice proportion following the different dynamics, we performed a Bayesian paired t-test in which we assign effect sizes a zero-centred Cauchy prior with scale 0.707. Of interest is the posterior distribution for the between-dynamic difference in choice proportion ΔCP_log_. The resulting posterior distribution is concentrated near a proportion difference of 0.23, with a central 95% credible interval for CP_log_ that ranges from 0.099 to 0.351. The null hypothesis states that agents will not change their choice proportion under different dynamical conditions, and thus predicts that the choice proportion will be equal for each condition (H0 ΔCP_log_ = 0). The alternative hypothesis is relatively informative and states that ΔCP_log_  is larger than 0, but that values of ΔCP_log_ close to 0 are more likely than values far above it (H+ : 1> ΔCP_log_ > 0) as seen in Fig 2I which shows the one-sided prior and posterior distribution for the effect size of ΔCP_log_ under H+. The paired Bayesian t-test revealed a Bayes factor of 52.376, which indicates the alternate hypothesis is around 50 times more likely than the null, which can be classed as very strong evidence. As shown in Fig 2I, compared to the prior distribution, the posterior distribution is concentrated near an effect size of 0.8. Robustness checks and sequential analyses can be seen in Fig 2J and 2K, and do not effectively change this interpretation. In conclusion, we find that gamble dynamics have a very strong effect on choice frequencies, with the gamble dynamics moving choice frequencies in the direction predicted by time optimality.


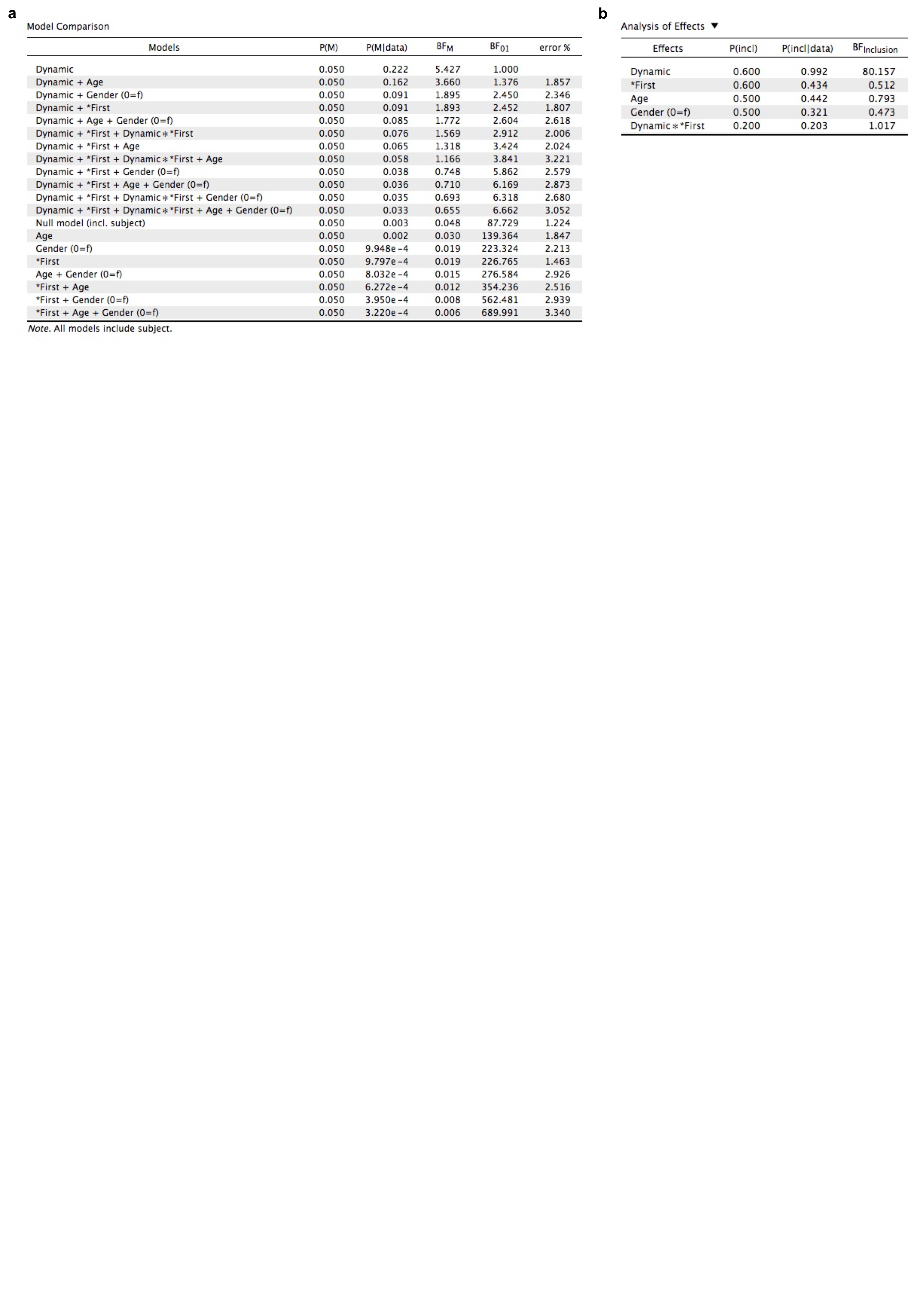


**S2 Fig | Model comparisons and analysis of effects for choice proportions. A,** the table of model probabilities, Bayes factors and error terms, for a repeated measures ANOVA on the choice proportions for discrepant trials. The meanings of each column are described in the text. **B,** the inclusion probabilities for all factors of interest across all models, along with the Bayes factors for their inclusion.

**Repeated measures ANOVA for choice proportions.** We conducted a Bayesian repeated measures ANOVA on the choice frequency data, with gender, age, and order of testing (*First) as between subject factors, and dynamic as within subject factors. We used the default prior options for the effects (r = 0.5 for the fixed effects, prior scale factor 0.707). To assess the robustness of the result, we also repeat the analysis over wide and ultrawide priors. The 'Model Comparison' table (S2A Fig in S1 Text) gives the results with respect to the different models that are compared. The models that are considered are all possible models including interactions of factors. The table lists all of the models, and the corresponding Bayes factors, where the best performing model (here, this is the model that includes only the dynamic factor) is compared to all the other models. The column of BF_01_ shows that the data are ~6.6 times more likely under the model with only the dynamic, than under the full model (i.e., the model with age, gender and order of testing, and their interactions). The column P(M) lists the prior model probabilities, which are held uniform across all the models. The column P(M|data) lists the posterior model probabilities. The column BF_M_ lists the comparisons between the best model (dynamic factor only) and each other model. The 'Analysis of Effects' (S2B Fig in S1 Text) gives Bayes factors for the inclusion of each effect that appears in at least one model. For each effect, the BF_inclusion_ column reflects how well the effect predicts the data by comparing the performance of all models that include the effect to the performance of all the models that do not include the effect. For the gamble dynamic, there is very strong evidence in favor of its inclusion (BF_inclusion_ > 80), whereas for all other factors there is either evidence against their inclusion, or only anecdotal evidence for their inclusion. In conclusion, compared to other factors and covariates, gamble dynamics have a uniquely strong effect on choice frequencies.


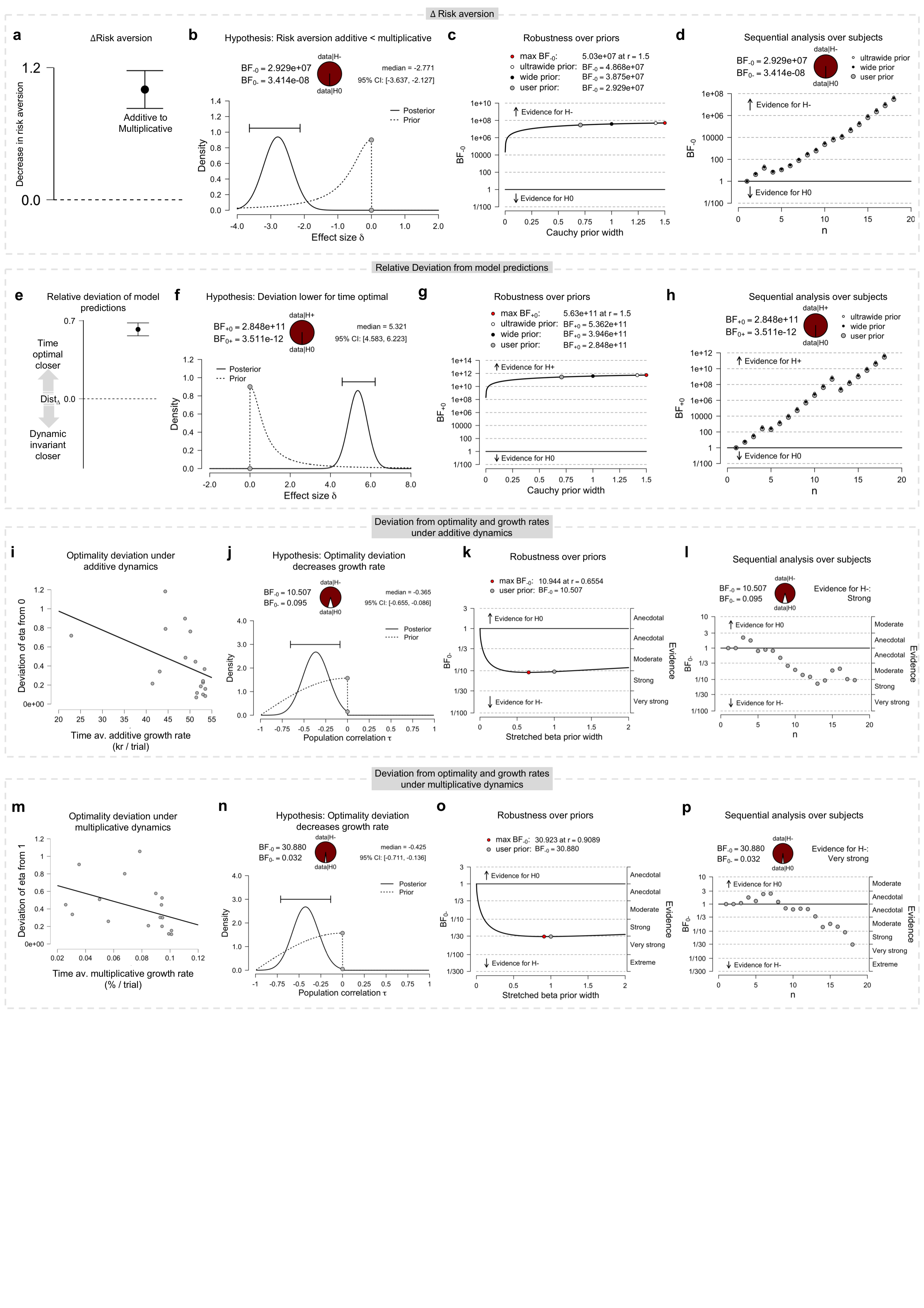


**S3 Fig | Descriptive statistics, priors & posteriors of hypothesis test, robustness tests and sequential analyses. A-D,** effect of dynamics on changing risk aversion parameters. **H-K,** comparison of the deviation of each model predictions of risk aversion parameters to those observed**. L-O,** effect of deviating from time optimality on the time average growth rates of subjects’ choices, under additive dynamics. **P-S,** equivalent effect under multiplicative dynamics.

**Gamble dynamics exert strong effects on risk aversion parameters.** To assess within subject changes in $\eta$ following the different dynamics, we performed a Bayesian paired t-test in which we assign effect sizes a zero-centred Cauchy prior with scale 0.707. Of interest is the posterior distribution for the between-dynamic difference in $\eta$, denoted Δ$\eta$. When comparing the $\eta$ of the multiplicative to the additive, the resulting posterior distribution is concentrated near a decrease of 1.01, with a central 95% credible interval for Δ$\eta$ that ranges from 0.829 to 1.172 (S3A Fig in S1 Text). The null hypothesis states that agents will not change their risk aversion under different dynamical conditions, and thus predicts that $\eta$ will be equal for each condition (H0: Δ$\eta$ = 0). The alternative hypothesis is relatively informative and states that Δ$\eta$ is less than 0, but that values close to 0 are more likely than values far below it (H- Δ$\eta$ < 0) as seen in S3B Fig in S1 Text which shows the one-sided prior and posterior distribution for the effect size of Δ$\eta$ under H-. The paired Bayesian t-test revealed a Bayes factor of 2.9 × 10^7^, which indicates extreme evidence in favour of the alternate hypothesis. As shown in S3B Fig in S1 Text, compared to the prior distribution, the posterior distribution is concentrated near an effect size of -3. Robustness checks over different prior widths can be seen in S3C and S3D Fig in S1 Text, and do not effectively change this interpretation. Descriptive statistics for the $\eta$ parameter are in S4A Fig in S1 Text. In conclusion, there are strong effects of gamble dynamics on risk aversion, with the multiplicative dynamics increasing estimated risk aversions, compared to additive dynamics.

**Estimates of risk aversion are closer to predictions of time optimal model.** To establish whether the $\eta$ values are closer in "$\eta$ -space" to the predictions of the time optimal or dynamic-invariant models, we computed the Euclidean distances of each subjects MAP estimate to each of the models predicted coordinate(s): In the time optimal case this is simply the distance to the [0,1] coordinate, whereas for the dynamic invariant utility model this is the shortest distance to the main diagonal (Fig 3C). We are interested to test whether these distances to the model predictions are smaller under the time optimal model, and thus we compute the difference in distance as Dist_Δ_ = Dist_invariant_ - Dist_time_. Dist_Δ_ had a mean of 0.65 (S3E Fig in S1 Text) indicating the time optimal model was closer in its predictions. To test this, we performed a Bayesian paired t-test in which we assign effect sizes a zero-centred half-Cauchy prior with scale 0.707. Of interest is the posterior distribution for the effect sizes of Dist_Δ_. The resulting posterior distribution is concentrated near a median effect size of 5.321, with a central 95% credible interval that ranges from 4.583 to 6.223 (S3F Fig in S1 Text). The alternative hypothesis is relatively informative and states that difference in distances will be positive, but that values close to 0 are more likely than values far above it (H+: Dist_Δ_ > 0) as seen in S3F Fig in S1 Text which shows the one-sided prior and posterior distribution for the effect size of Dist_Δ_ under H+. The null hypothesis states that the distance of the data to the predictions is larger for the dynamic invariant model than for the time optimal model, and thus predicts that the difference in distances will be negative (H0 : Dist_Δ_ < 0). The paired Bayesian t-test revealed a Bayes factor of 2.8 × 10^11^, which indicates extreme evidence in favour of the alternate hypothesis. As shown in S3F Fig in S1 Text, compared to the prior distribution, the posterior distribution is concentrated near an effect size of 5.3. Robustness checks and sequential analyses over different prior widths can be seen in S3G and S3H Fig in S1 Text, and do not effectively change this interpretation. In conclusion, there is extreme evidence that the estimated risk aversions are closer to the prediction of the time optimal model than a model which assumes no dynamic specific changes in risk aversion.

**Deviations from time optimality correlates negatively with time average growth rates.** We conducted a Bayesian correlation analysis for the relation between the deviation of the estimated risk aversion from time optimality, and the time average growth rate achieved by the participants’ choices. We used a default prior (as specified in JASP software) which yields a uniform distribution on Kendall's $\tau$^3^. We focus on hypothesis testing, specifying a one-sided alternative hypothesis which posits a negative correlation between deviation from time optimality and time average growth rate, compared to the null hypothesis that postulates that the correlation is non-negative. The Bayes factor for each correlation quantifies the evidence in favor of a negative correlation. Negative correlations were found for both additive dynamics (BF_-0_ = 30.88, BCI_95%_ [-0.656, -0.068]) and multiplicative dynamics (BF_-0_ = 10.51, BCI_95%_ [-0.711, -0.131]). These correlations yielded BF_-0_ > 10, indicating strong evidence in favor of the alternative hypothesis that postulates the presence of a negative correlation. The posterior distributions for each of the correlations are in S3J and S3N Fig in S1 Text, and scatterplots between the variables are in S3I and S3M Fig in S1 Text, including a fitted linear regression line. Robustness checks and sequential analyses over different prior widths are shown in S3K, S3L, S3O, and S3P Fig in S1 Text. A table of statistics, including $\tau$estimates is in S4D Fig in S1 Text.


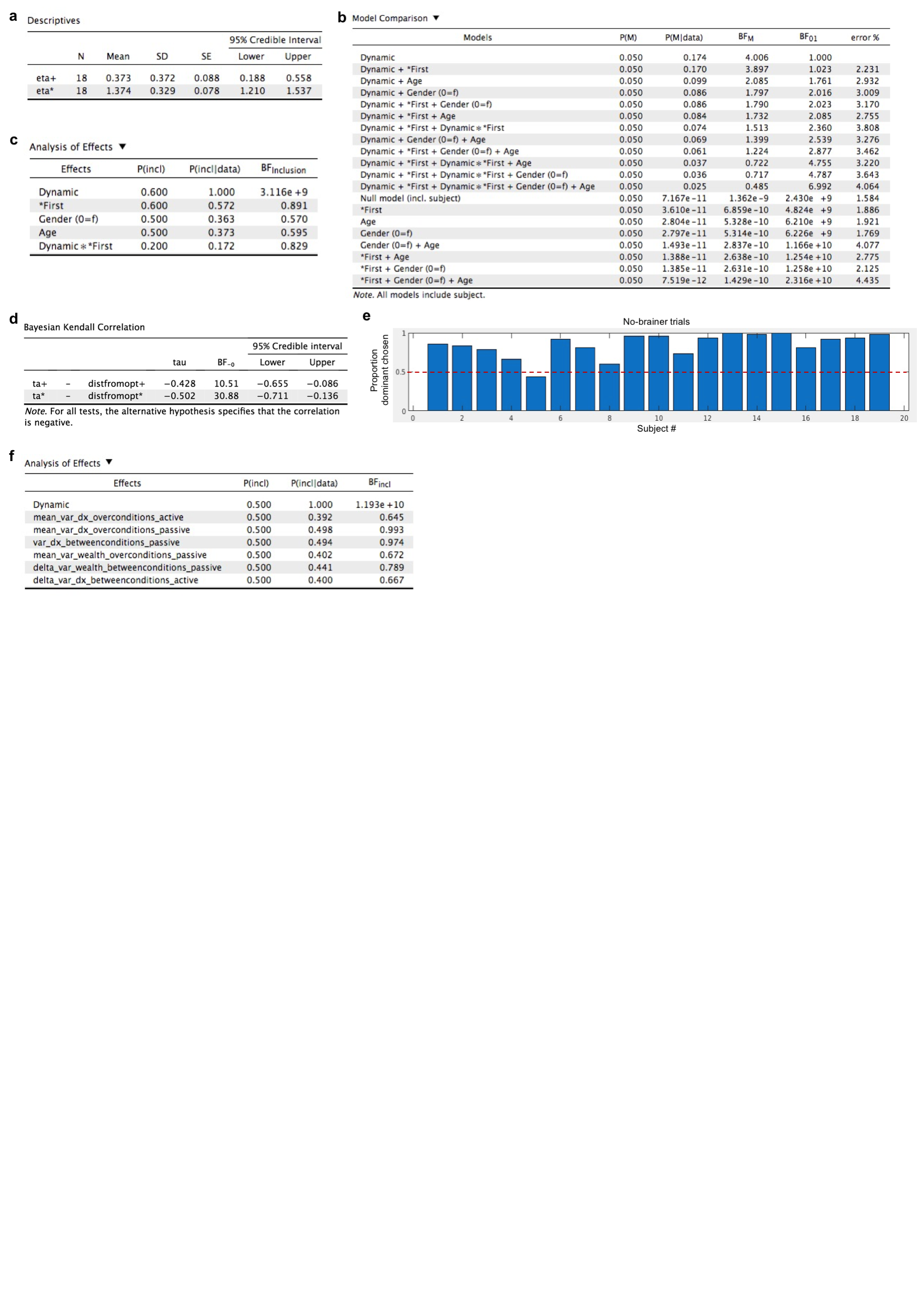


**S4 Fig** | **Tables and no-brainers. A,** descriptive statistics for the risk aversion parameter, $\eta$. SD -standard deviation; SE - standard error mean. **B,** table of models compared in repeated measures ANOVA for risk aversions. Column headings described in main text. **C,** table of Analysis of effects shows the Bayes factors for the inclusion of each factor across all of the models in b. P(incl) indicates the prior probability of each effect across all models. BF_Inclusion_ is the Bayes factor for the inclusion of that factor, comparing all models with vs. without that factor. **D,** table for Kendall correlation, where correlations between time averages (ta) and distances from optimal risk aversion parameters ('distfromopt') are tabulated. **E,** proportion of correct responses (dominant chosen) in the no-brainer trials. Red line indicates chance performance. **F,** Analysis of effects shows Bayes factors for the inclusion of each factor, including both the dynamic, and the covariates derived from the variances in wealth and changes in wealth.

**Repeated measures ANOVA shows strong effect of gamble dynamics on risk aversion.** We conducted a Bayesian repeated measures ANOVA on the risk aversion parameter $\eta$, with gender, age, and order of testing as between subject factors, and dynamic as a within subject factor. We used the default prior options for the effects (r = 0.5 for the fixed effects). To assess the robustness of the result, we also repeat the analysis for different widths of prior. The 'Model Comparison' table in S4B Fig in S1 Text gives the results with respect to the different models. The models that are compared are all possible combinations of factors including interactions of factors. The table lists all of the models, and the corresponding Bayes factors, where the best performing model (here, the model that includes only the dynamic factor) is compared to all the other models. The column of BF_01_ shows that the data are ~7 times more likely under the model with only the dynamic, than under the full model (i.e., the model with age, gender and order of testing, and their interactions). The column P(M) lists the prior model probabilities, which are held uniform across all the models. The column P(M|data) lists the posterior model probabilities. The column BF_M_ indicates how many times the best model is compared to each other model. The 'Analysis of Effects' (S4C Fig in S1 Text) gives Bayes factors for the inclusion of each factor that appears in at least one of these models. For each factor, the BF_Inclusion_ column reflects how well the effect predicts the data by comparing the performance of all models that include the factor to the performance of all the models that do not include the factor. For the factor representing the gamble dynamic (Dynamic), there is extreme evidence in favor of its inclusion (BF_Inclusion_ > 100), whereas for all other factors there is evidence against their inclusion. In conclusion, compared to other factors, the gamble dynamic has a uniquely strong effect on risk aversions.

**Idiosyncratic wealth trajectories exert no systematic effect on risk aversions.** Since the passive phase was designed to have stochastic paths, subjects can end the passive phase with different wealths, and also have experienced different volatilities (variance of wealth changes). We tested whether these differences between both subjects and conditions, could account for the differences in risk aversions observed. To this end, we performed a rmANOVA with eta as dependent variable, and dynamic as repeated measures factor. The following subject-wise covariates were added for changes in wealth: mean variance across both conditions for passive phase ('mean_var_dx_overconditions_passive'); difference in variance between conditions for passive phase ('delta_var_dx_betweenconditions_passive'); mean variance across both conditions for active phase ('mean_var_dx_overconditions_passive'); difference in variance between conditions for active phase ('delta_var_dx_betweenconditions_passive'). Two further subject-wise covariates were added for in-game wealth itself: mean variance across conditions for passive phase ('mean_var_wealth_overconditions_passive'); difference in variance between conditions for passive phase ('delta_var_wealth_betweenconditions_passive'). Note that S4E Fig in S1 Text shows that the Bayes Factor for the inclusion of the dynamic factor is extreme, whereas all other covariates are below 1. This indicates that there is not even moderate evidence that adding wealth covariates to the model improves its predictive adequacy.


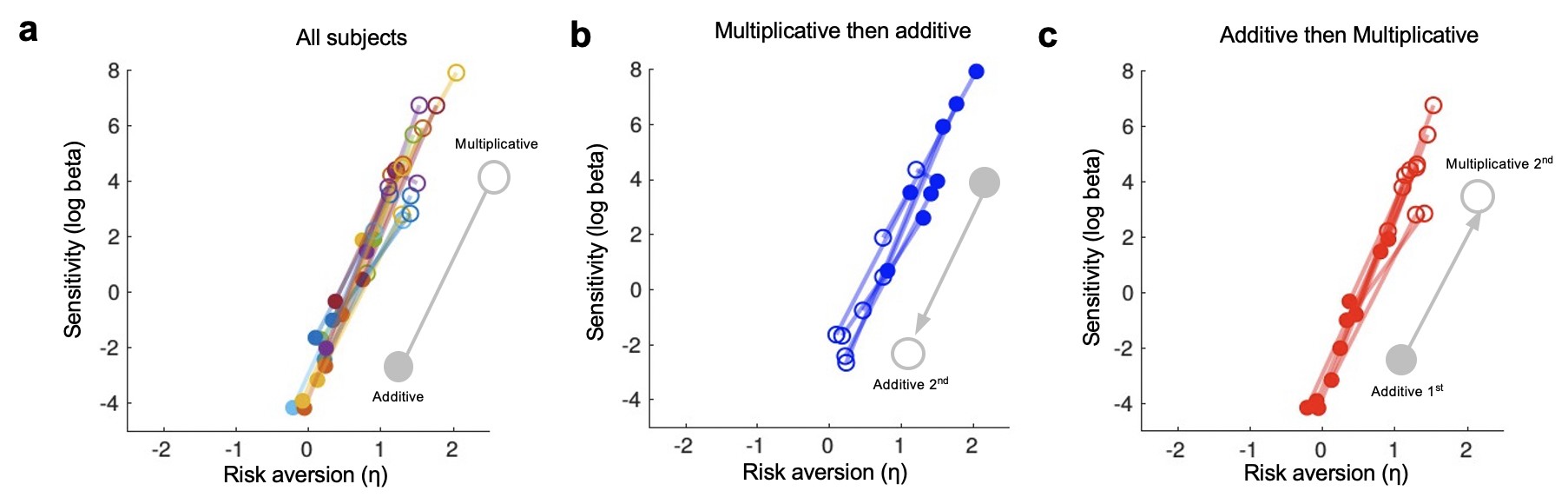


**S5 Fig** | **Displacements of model parameters as a function of dynamics. A,** displacement in parameter space caused by changing the gamble dynamic. Filled and empty circles indicate additive and multiplicative dynamics, respectively. **B-C,** equivalent displacements splitting subjects according to the temporal order of their experience of the dynamics.


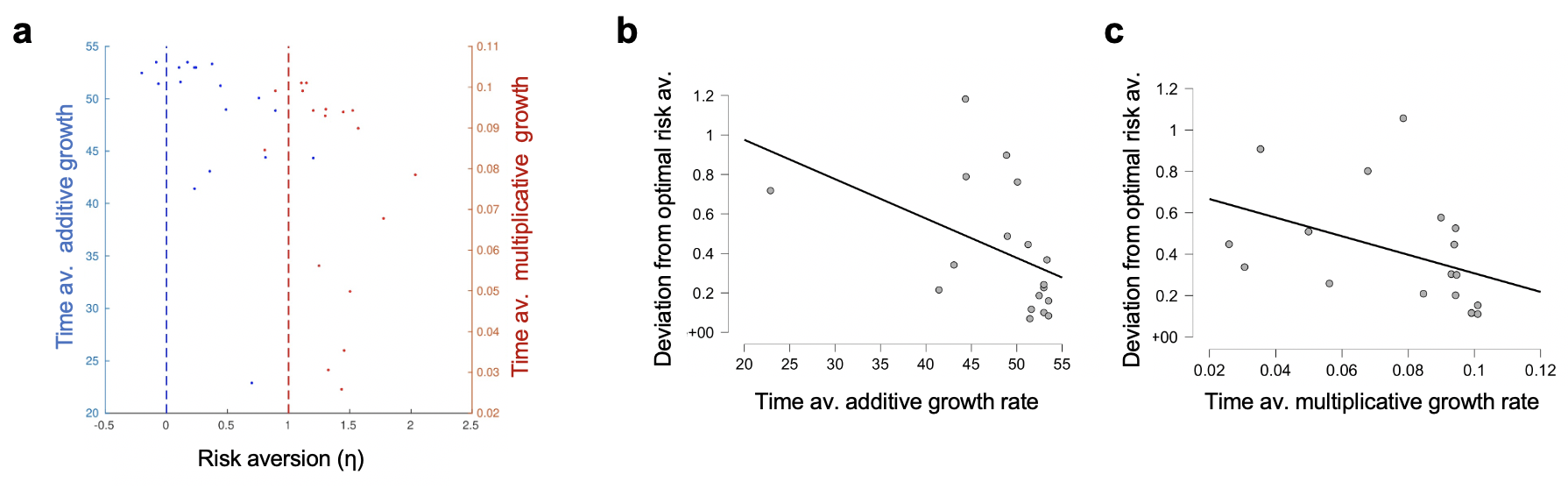


**S6 Fig** | **Time average growth rates as a function of risk aversion parameters. A,** distribution of subject specific time average growth rates and risk aversion under both dynamics. **B,** correlation between time average additive growth rate of subject's choices and deviation of subject's risk aversion away from the time optimal value. **C,** equivalent plot for multiplicative dynamics.

**Parameter recovery.** To evaluate whether the model estimation methods were capable of recovering approximate parameter estimates, we performed a parameter recovery simulation in which we subjected our estimation procedures to synthetic data for which ground truth parameter values were set a priori. S7A Fig in S1 Text shows the correspondence between the estimates of risk aversion parameters and the ground truth values used in simulating synthetic agents. Agents were simulated to have all pairwise combinations of $\eta$ values of [-0.5,0,0.5,1,1.5] for additive and multiplicative dynamics. 20 subjects were simulated for each parameter combination, and then the same parameter estimation procedures were applied to visualise the recovery of parameters as used in Fig 3. This includes the estimation of both $\beta$ and $\eta$ parameters. S7A Fig in S1 Text shows a subset of this space most relevant to the key results of this paper. The fact that $\eta$ can be recovered accurately shows that it cannot be adequately captured via other parameters such as the sensitivity parameter $\beta$. This is also evident from the relative precision of the posterior $\eta$ values observed in the original data in Fig 3.

**Model recovery.** To evaluate whether the model selection methods were capable of recovering the set of utility models tested, we performed a model recovery simulation in which we subjected our estimation procedures to choices made by synthetic agents for which ground truth model values were set a priori. S7B Fig in S1 Text shows the correspondence between the posterior inclusion probabilities for each utility model and the ground truth identities of the utility models used in simulating synthetic agents. The first seven subjects were synthesised as prospect theory agents (with same parameters for additive and dynamic sessions), the next seven subjects as isoelastic utility agents (again with same parameters for both sessions), and finally the last seven subjects were time optimal agents.


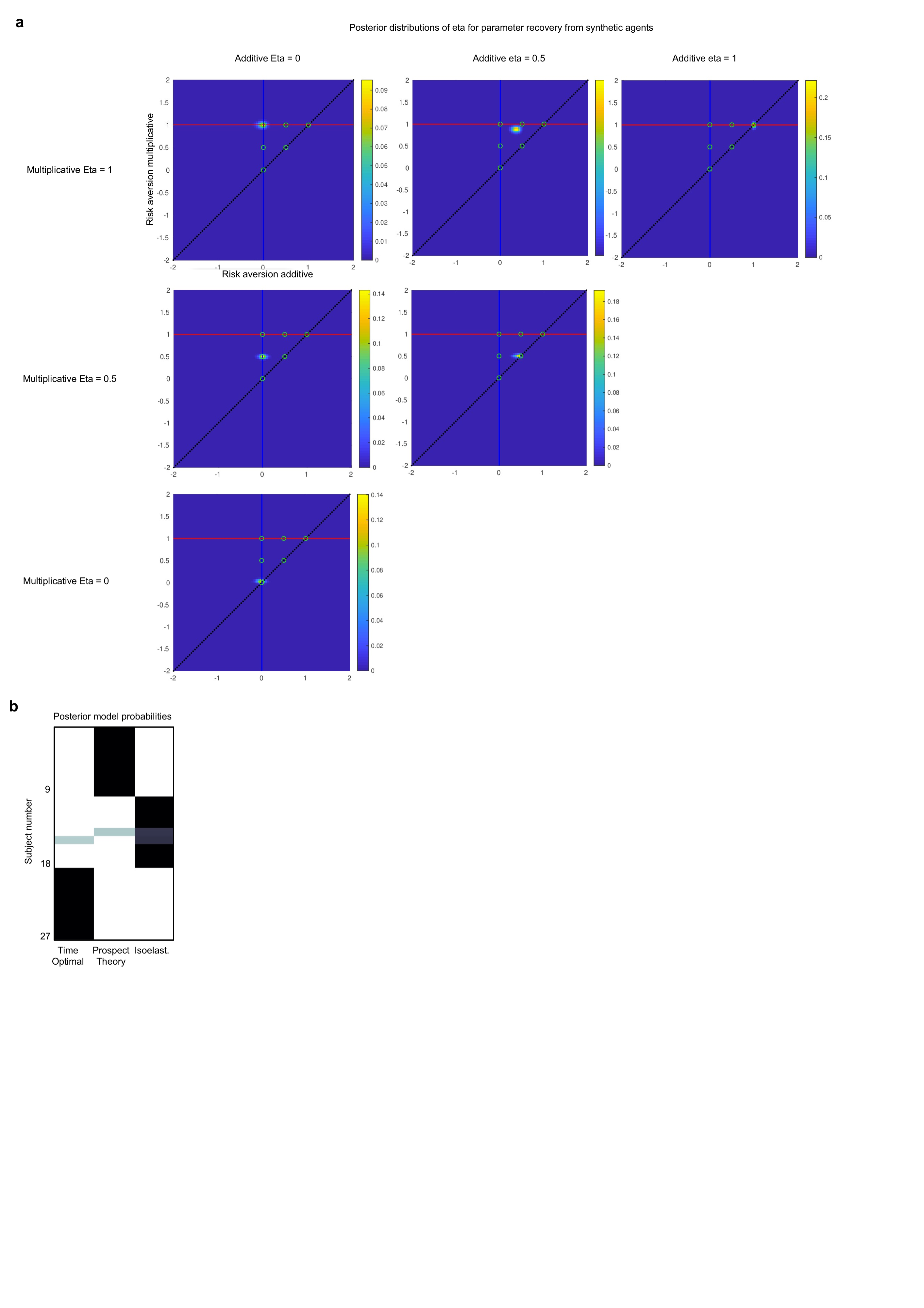


**S7 Fig** | **Parameter and** **Model recovery. A,** parameter recovery for several populations of synthetic agents with different combinations of $\eta$ parameters. Each panel shows the posterior $\eta$ distribution, marginalised over subjects, estimated via the same model and code as the real data shown in Fig 3. **B,** model recovery for three different groups of synthetic agents, for the three models compared. Color range shows posterior model probabilities as in Fig 4C (black = 1, white = 0). Posterior model probabilities map strongly onto the ground truth model identities, insofar as the first nine agents were synthesised via a time optimal model, the next nine from a prospect theory model, and the final nine from an isoelastic utility model. Note that the isoelastic agent obtains small posterior model probabilities in the range of ~0.05 for the two other models, for only 2/9 parameter values.

**References**

1. Ly, A., Verhagen, J. & Wagenmakers, E.-J. Harold Jeffreys’s default Bayes factor hypothesis tests: Explanation, extension, and application in psychology. *Journal of Mathematical Psychology* **72**, 19–32 (2016).

2. Jeffreys, H. *Theory of probability*. (Clarendon, 1961).

3. van Doorn, J., Ly, A., Marsman, M. & Wagenmakers, E.-J. Bayesian Inference for Kendall’s Rank Correlation Coefficient. *The American Statistician* **72**, 303–308 (2018).
