# Supplementary material for: Ergodicity-breaking reveals time optimal decision making in humans
Source: PLoS Comput Biol. 2021 Sep 9;17(9):e1009217. doi: 10.1371/journal.pcbi.1009217 (PMC8454984; doi:10.1371/journal.pcbi.1009217)
Supplement: S2 Text — (DOCX) [file pcbi.1009217.s002.docx]

**S2­ Text**: **Synthetic agents**

Figs 1E and 1F shows wealth trajectories of synthetic agents repeatedly playing the set of 144 different gambles used in this paradigm. No-brainers were not included. 12 different agents were synthesised, comprising the three model classes: 9 variants of prospect theory agents, comprising all possible combinations of $\lambda$ {1, 2, 3} and α {0.3, 0.6, 0.9} (identical for both gains and losses), and 2 variants of isoelastic agents η {0, 1}. Each prospect theory and isoelastic agent had the same parameters for both additive and multiplicative dynamics. The time optimal agent is a special case, having linear utility under additive dynamics, and logarithmic utility under multiplicative dynamics. The trajectories were computed over several timescales, hour, day, week, year. At a long enough timescale noise is removed by the passage of time, and the time average growth rates of the different agents become apparent. In this artificial environment, agents were playing trials every 9.5s, continuously. Over the duration of a week, a reasonable approximation to the time average growth rate is typically revealed (Figs 1E and 1F).
