# Supplementary material for: Ergodicity-breaking reveals time optimal decision making in humans
Source: PLoS Comput Biol. 2021 Sep 9;17(9):e1009217. doi: 10.1371/journal.pcbi.1009217 (PMC8454984; doi:10.1371/journal.pcbi.1009217)
Supplement: S3 Text — S1 Fig in S3 Text. State-space graphs for different versions of the task. A, state-space graph of a simplified version of the game comprising only two possible gamble pairs, and with only two trials. B, the same state-space for four trials. C, The state-space for this experiment for a single trial. In all graphs A, B and C-, red dots indicate branches between possible pairs of gambles, yellow dots indicate possible branches between choices, and green dots indicate branches between different possible outcomes. (DOCX) [file pcbi.1009217.s003.docx]

**S3 Text: Supplementary Discussion**

**House money.** Another validity issue for economic experiments is the issue of “house money”, which pertains to whether subjects behave differently if they perceive the money not to be entirely theirs^1^. These effects are typically circumvented by the pre-endowment of money, or by having subjects work in order to feel ownership of the money. Both of these strategies were implemented here, insofar as subjects knew days ahead of their upcoming endowment, and they were pre-endowed approximately 90 minutes prior to decision-making. The sense of ownership was enhanced by subjects actively causing changes in this wealth via 338 button presses and observing the resulting fluctuations for a total of ~60 minutes. Further, house money as a putative effect does not explain the condition-specific changes in risk aversion observed, nor their bivariate alignment with the time optimal strategy (Fig 3E).

**Cognitive considerations.** It is notable that subjects were able to perform the task under challenging cognitive conditions. Gambles were chosen based on the participant's memory of the stimuli from the previous passive session, up to 60 min ago, with choices being made every ~10s for ~1 hour of testing per day in a noisy environment. Nearly all participants (18 of 19) tested could choose dominant gambles above chance in the No-brainer trials (S4E Fig in S1 Text). The degree to which subjects could approximate the time optimal strategy indicates that they had a relatively high-fidelity magnitude representation of the underlying growth increments and factors. Though time optimality was a reasonable approximation of the observed data, the estimated risk aversion parameters were systematically biased to be more risk averse than predicted by time optimality (Fig 3P). This may be due to noise in the sensory or mnemonic encoding of the stimuli. Such sources of noise may increase the apparent risk aversion due to the uncertainty it adds to each gamble.

**Dynamical utility models.** The models tested so far were all static, in the sense that they do not incorporate any anticipation of future gambles or wealth trajectories. This is because the game is effectively a single period game in which the 10 randomly selected outcomes are realised at once at the end of the game, with no intermediate wealth updating. Nevertheless, one strategy that subjects could take is to plan ahead, making decisions that maximise the expected utility of the terminal wealth occurring at the end of the game. This is predicted under multi-period expected utility theory^2-4^ which involves an iterative evaluation of wealth computed via dynamic programming. Such models compute all terminal wealths that are possible under the different contingencies, and work backwards to derive the optimal choices, given the agent’s utility function. However, such a strategy is not possible for subjects in this game. This is because the branching factor for each trial is extreme (>600 per trial, S1C Fig in S3 Text), and the subject lacks critical information necessary to compute terminal wealths, such as knowing what the space of possible gamble pairs is, or even knowing the total number of trials they will face. We show that taking into account even optimistic estimates of cognitive constraints, results in search horizons that are so myopic, that the predictions of multiperiod models are scarcely different from the static versions.

**Dynamic versions of expected utility theory and prospect theory.** Multiperiod EUT models assume a single utility function for evaluating the terminal wealths, which is invariant across all settings. The subtlety is that, an experimenter estimating utility functions based on the observed choices of a multiperiod EUT agent would reveal an estimated utility function that appears to change between different dynamical settings, even though the agent is optimising the same utility function for terminal wealths. Indeed, this was proposed as a candidate model for this experiment^5^ on the basis that it can be shown that risk aversion can decrease in the context of repeated additive gambles, when compared to multiplicative gambles. This will be important for evaluating expected utility models under dynamical settings, however multiperiod EUT is not a viable model of human behavior for this experimental task. Firstly, the terminal wealths are not tractable. This is because the subjects did not know the following five details necessary to perform a backward induction from terminal wealths: a) how many gambles they would face; b) what gambles they will face; c) the outcome of each gamble; d) which choices would be realised; e) what their current wealth is at the time of choosing. Secondly, even if we were to imagine that these uncertainties are not a problem for computing terminal wealths, the combinatorics of the game are computationally prohibitive, as we will show in the next two sections.

**A simplified game tree.** To illustrate the forking possibilities within the experiment, consider a much simpler experimental game, involving only two possible pairs of gambles, lasting only two trials long. Here the graph of the state space looks like S1A Fig in S3 Text. From the central node, the graph branches first into two, one for each of the possible pairs of gambles (red dots), then it branches in two again for the binary choice that the agent makes (yellow dots), then it branches again to determine which of the two stimuli are realised by the fair coin (green dots). After one trial there are thus 8 possible terminal wealths (inner green circle of points), and after two trials there are 64 (outer green circle). Evaluating trees via planning is computationally expensive in terms of time, working memory, and metabolic energy. There is however some evidence humans can partially search trees of this size via a model-based cognitive system that evaluates the state based contingencies of sequences of choices^6,7^. On the available evidence, performance in tree-based search appears to fall off steeply with depth, with depths of 5 (for branching factors of 2) being the point at which chance performance is reached for discriminating best outcomes^6^. In the simplified game illustrated in S1A Fig in S3 Text, subjects would be at chance performance by the time they are evaluating the terminal wealths after only two trials. According to the calculations of Goldstein^5^, the change in the risk aversion parameter for a subject with logarithmic utility playing a repeated additive game, searching ahead two trials would result (for his setup) in approximately a shift of only 2% down to 0.98. This level of change in risk aversion is not sufficient to account for the changes in risk aversion that were observed (Fig 3L). With such myopic search capacities, then the multiperiod model proposed by Goldstein^5^ result in changes in risk aversion that are on the scale of those already illustrated in Fig 3C (lower). The computational overheads grow exponentially with search depth. For instance, to search 4 trials ahead, S1B Fig in S3 Text shows that 1536 terminal wealths must be evaluated. Even if it were cognitively possible to evaluate this many nodes of a tree, which we assert it is not, then risk aversion would drop by only ~5%.

**The actual game tree for this experiment.** If we are to temporarily assume, what we know is impossible, that subjects know exactly the unknown gamble space and the number of trials, then the state space graph looks like that of S1A Fig in S3 Text. This graph has a per trial branching factor with a lower bound of ~600 and a depth of 312. By comparison, the game Go has a branching factor of ~200 per move and an average depth of ~200. Evidence from neural decoding as subjects engage in planning tasks, suggest that the fastest state to state transitions are on the order of 40ms, which would correspond to approximately 25 evaluations per second^8^. Given that subjects have ~6 seconds between trials to engage in search, the number of graph nodes that can be evaluated per trial is well below what is necessary to plan one trial ahead. Even if subjects could summon cognitive capacities surpassing what has been previously observed, this would again result in only minor modulations to the risk aversions predicted by the static models. Finally, and perhaps most importantly, since multiperiod EUT is an extension to expected utility theory, it does not yield quantitative prediction for which utility functions the agent should be maximising at terminal wealth. On these considerations, multiperiod EUT is ill-suited to games of this scale, complexity, and epistemic uncertainty. Even if such issues are to be resolved or dismissed, they still do not provide quantitative predictions compatible with the data observed here. We also note that whilst it is possible to derive dynamical versions of prospect theory^9,10^, the same fundamental limitations apply to these models. In both cases the cognitive limits on planning ahead make the predictions of the dynamic and static versions effectively the same.

**
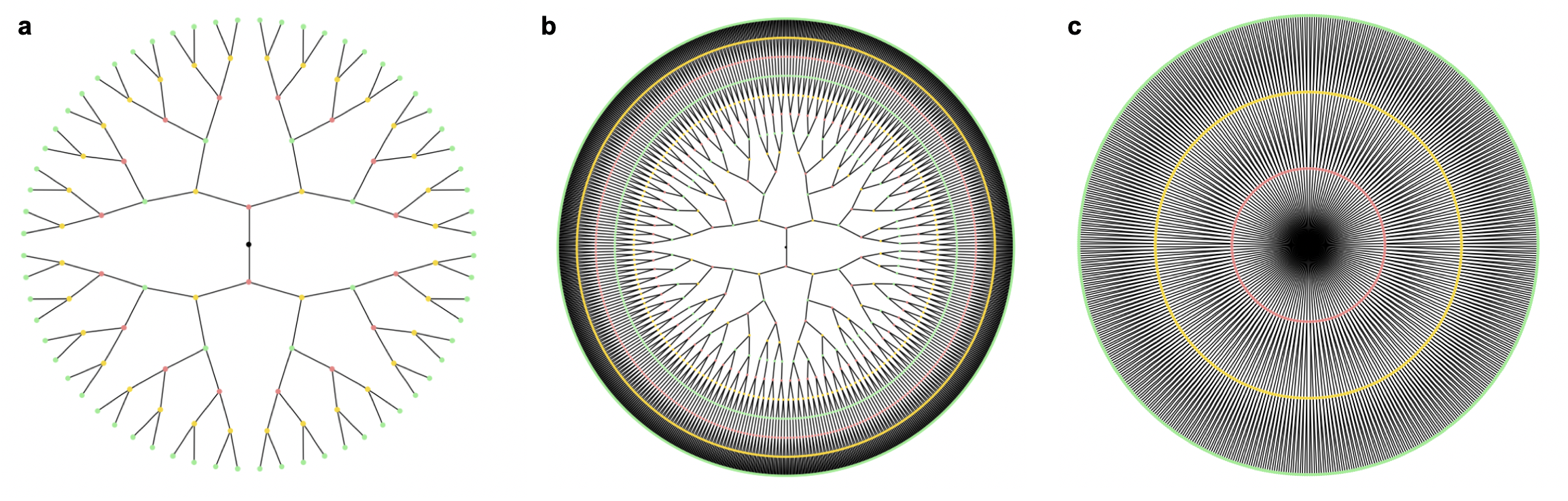
**

**S1 Fig** | **State-space graphs for different versions of the task. A,** state-space graph of a simplified version of the game comprising only two possible gamble pairs, and with only two trials. **B,** the same state-space for four trials. **C,** The state-space for this experiment for a single trial. In all graphs A, B and C-, red dots indicate branches between possible pairs of gambles, yellow dots indicate possible branches between choices, and green dots indicate branches between different possible outcomes.

**References**

1. Thaler, R. H. & Johnson, E. J. Gambling with the House Money and Trying to Break Even: The Effects of Prior Outcomes on Risky Choice. Management Science 36, 643–660 (1990).

2. Merton, R. C. Lifetime Portfolio Selection under Uncertainty: The Continuous-Time Case. The Review of Economics and Statistics 51, 247 (1969).

3. Samuelson, P. A. Lifetime Portfolio Selection By Dynamic Stochastic Programming. The Review of Economics and Statistics, 51 (3), 239-246 (1969).

4. Mossin, J. Optimal Multiperiod Portfolio Policies. The Journal of Business 41, 215–229 (1968).

5. Goldstein, A. Did Ergodicity Economics and the Copenhagen Experiment Really Falsify Expected Utility Theory? Researchers.one, <https://researchers.one/articles/20.02.00002v1> (2020)

6. Huys, Q. J. M. et al. Bonsai Trees in Your Head: How the Pavlovian System Sculpts Goal-Directed Choices by Pruning Decision Trees. PLoS Comput Biol 8, e1002410 (2012).

7. Keramati, M., Smittenaar, P., Dolan, R. J. & Dayan, P. Adaptive integration of habits into depth-limited planning defines a habitual-goal–directed spectrum. PNAS 113, 12868–12873 (2016).

8. Kurth-Nelson, Z., Economides, M., Dolan, R. J. & Dayan, P. Fast Sequences of Non-spatial State Representations in Humans. Neuron 91, 194–204 (2016).

9. He, X. D. & Zhou, X. Y. Portfolio Choice Via Quantiles. Mathematical Finance 21, 203–231 (2011).

10. Barberis, N. A Model of Casino Gambling. Management Science 58, 35–51 (2012).
