# Supplementary material for: Ergodicity-breaking reveals time optimal decision making in humans
Source: PLoS Comput Biol. 2021 Sep 9;17(9):e1009217. doi: 10.1371/journal.pcbi.1009217 (PMC8454984; doi:10.1371/journal.pcbi.1009217)
Supplement: S4 Text — (DOCX) [file pcbi.1009217.s004.docx]

**S4 Text: Reply to Doctor, Wakker, and Wang**

The following is a reply to comments made by Doctor and colleagues (2020)^1^ on a pre-print version of this paper. We include quotations in full from the appendix of the reply paper (removing references for clarity):

*“Meder et al. applied expected utility and prospect theory in a way that we call static: they applied EU and PT to each choice in each round separately, as if it was the only choice made and as if intermediate outcomes were actually received. This static analysis is incorrect. The intermediate outcomes are not outcomes received and consumed by subjects. Instead, they are only intermediate values playing a complex role in determining the final outcome, which is the only outcome received by the subject. A dynamic analysis should have been used, explained next.”*

Dynamic analyses are incapable of performing the experimental task as we argue in section “Dynamical utility models” in the Supplementary Discussion. In short it is effectively impossible, given the informational and cognitive constraints of the subject to look ahead more than one trial ahead. If subjects are limited by experimental design, unable to compute outcomes of the future, the dynamic models, when faced with this task, effectively make the same predictions as the static models.

*“This means applying EU to terminal wealth, which in this experiment concerns final outcomes. PT is to be applied to perceived changes in wealth, which in this experiment also refers to final outcomes. It was made very clear to the subjects that the final outcome was the only one they would receive, and that the intermediate outcomes were only components to determine the real, final, outcome.”*

*“For a normative analysis of EU with full information about the experimental procedure, a sophisticated probability calculus should be carried out to determine for each sequence of 312 choices what probability distribution over final outcomes is degenerated by that combination of choices. As explained before, this is a probability distribution over the interval [0, 560]. Then, the combination of choices with the highest EU is chosen.”*

A normative analysis with full information is not informative as a model of what subjects should do, with the limited information that they have. For a normative analysis to be useful in interpreting the data, it should have the same or at least similar informational constraints as the subjects. Just as it would be absurd to assume that subjects can access the state of the computer to pick whichever gamble will randomly payout, it is equally absurd to interpret a model which knows how many trials there are, what the future gamble choices will be, which gambles will be realized, and so on. This is what we are being invited to entertain in this normative analysis.

*“For a descriptive analysis using EU or PT that seeks to describe what subjects actually did in the experiment (the case of interest here), it is unrealistic to assume that subjects can determine the probability distributions over final outcomes that are generated by their choices. This would be too cognitively demanding under full prior information, and in reality is impossible because subjects do not know precisely what stimuli are to come in advance. Hence, subjects face a situation of unknown probabilities, often called choice under ambiguity in economics. They may (as-if) have considered sets of possible probability distributions over [0, 560], and used maxmin expected utility, or any other of the modern ambiguity theories, which we will not elaborate on here.”*

This is predicated on the assumption that they can link their behavioral strategy, to the sets of probability distributions over 300 trials later. If they cannot compute ahead more than 1 trial even this possibility is foreclosed. They do however know what gamble is presented to them and they know the explicit probabilities of payout of each fractal with certainty. The time optimal strategy is available to them if they know the dynamic and have some implicit understanding of the ergodicity transformation. Based on the results observed, this explains the results with greater specificity and with far greater parsimony.

*“Treatment is much harder to assess than treatment+ (see the aforementioned exponential growth bias), therefore carrying more ambiguity. Hence, ambiguity aversion (risk aversion for unknown probabilities) will be considerably greater there. This can explain the greater aversion found in treatment, and confounds the claims on utility curvature by Meder et al. and Peters (2019).”*

We are open to the possibility that there are differences between the conditions, this is indeed discussed in the main discussion, section “Differences between conditions”. It is possible there is greater uncertainty about the effect of the different fractals in the multiplicative condition, and this could cause a greater risk aversion (RA). This claim is in effect a model which states that there is an inequality constraint such that RA_X_ > RA_+_ . This predicts that risk aversion parameters should sit in the upper triangle of Fig 3C. This is not a specific prediction because it is still compatible with half of the parameter space. It might be true, but the establishment of this truth is not awarded much credence in the model because its predictions were so vague. It is analogous to correctly predicting the location of a submarine to be in the northern hemisphere. Instead the time optimal theory predicts both the inequality and the approximate bivariate location of the risk aversion parameters. To return to the same analogy, it would be as if the submarine were predicted to be in European waters, and that this was subsequently shown to be true. It is for this reason that, given the data we observed, the time optimal model is guaranteed to predictively outperform any ambiguity model that only predicts the direction of an inequality. In other words, the ambiguity model, at least as articulated by Doctor and colleagues cannot explain why the parameter estimates for the risk preferences appear to approximate the time optimal strategy. If another model is to be seriously proposed as an alternate explanation, it should be able to specifically predict the data as well as the model it seeks to topple or better. The ambiguity account does not come close to doing this.

*“Further, increased complexity of probability calculus in itself increases aversion, again, irrespective of utility curvature. Thus, the strong focus of economics on psychology and the full consideration of dynamics, as properly done in any economic analysis, leads to a qualitative explanation of the differences found.”*

The prediction of an order constraint between two conditions, made after the data has been observed, is not much of a prediction, nor is it much of an explanation. It contains no quantitative explanation, which would be substantially more vulnerable to falsification. To repeat, the time optimal mode predicted the bivariate location of the risk preference parameters, which proved to be a reasonable approximation, and importantly, substantively better than other models that could perform the task. The ambiguity model simply predicted which half of the parameter space it will be in. Again, based on the data so far, the time optimal model has a superior predictive performance.

*“Peters was overly negative when writing: “the strong focus on psychology and lack of consideration for dynamics, prevalent in expected utility theory, corresponds to the belief that the difference between the red and blue curves is spurious”. Because both dynamics and uncertainty are central in Meder et al.’s stimuli, any model focusing on only one of these two will be deficient. Ergodic economics and expected utility should not compete but should collaborate here.”*

We openly invite decision scientists and economists to collaborate with us on a future replication of this study, including Doctor, Wakker and Wang. Pre-registration of how the ambiguity model would play the paradigm, along with any other dynamic utility model, or any other established model proposed by the decision science community, is very much our ambition for future experiments.

*“Note that equation (10) in Meder et al. does not capture prospect theory because probability weighting is omitted. This equation (10) is in fact a special case of EU with a different, three-parameter, utility function. Even if all probabilities are 0.5, then still probability weighting is crucial and does not cancel, contrary to some claims to the opposite. There is more risk aversion as 𝑤(0.50) is smaller, which should be corrected for before estimating utility. For instance, with, for simplicity, linear utility, the lottery (0.5: 90, 0.5: 10) is evaluated by 𝑤(0.5) × 90 + (1 − 𝑤(0.5)) × 10, and* *the lottery (0.5: 60, 0.5: 40) is evaluated by 𝑤(0.5) × 60 + (1 − 𝑤(0.5)) × 40 (equation 220; p. 30127; Appendix 9.810). There is a strict preference for* *(0.5: 90, 0.5: 10) over (0.5: 60, 0.5: 40) if 𝑤(0.5) > 0.5, but the preference reverses if 𝑤(0.5) < 0.5. This shows that probability weighting 𝑤 cannot be ignored even if all probabilities are 0.5. Further, the total money accumulated up to that point, 𝑌, cannot be ignored in the utility calculations. Note that loss aversion plays no role in this experiment because final outcomes can never be losses.”*

Probability weighting is omitted because it is assumed to cancel out if the *w*(0.5) is the same, or approximately the same, for both gains and losses. Since most of the experimental literature on probability weighting does not obtain a separate probability weighting function for gains and losses^2^ the claim that there should be separate parameters to account for gains versus losses is not empirically motivated. It is not unusual to set the same probability weighting to both gains and losses, nor is there presently an empirical motivation to do so, and indeed is a practice upheld by at least one of the authors of the present critique^3^. That said, we appreciate Doctor and colleagues alerting us to this variant, and we were able to amend this paper such that readers are aware that some versions of PT would have different weighting functions for gains and losses.

**References**

1. Doctor JN, Wakker PP, Wang TV. Economists’ views on the ergodicity problem. Nat Phys, (12):1168–1168 (2020)

2. Verschoor A, D’Exelle B. Probability weighting for losses and for gains among smallholder farmers in Uganda. Theory Decis. https://doi.org/10.1007/s11238-020-09796-8

(2020)

3. Fennema H, Wakker P. Original and cumulative prospect theory: a discussion of empirical differences. Journal of Behavioral Decision Making. 1;10(1):53–64 (1997)
