# Supplementary material for: Ergodicity-breaking reveals time optimal decision making in humans
Source: PLoS Comput Biol. 2021 Sep 9;17(9):e1009217. doi: 10.1371/journal.pcbi.1009217 (PMC8454984; doi:10.1371/journal.pcbi.1009217)
Supplement: S5 Text — (DOCX) [file pcbi.1009217.s005.docx]

**S5 Text: Experimental checklist**

**Before subject arrives**  ☐

Get paperwork (subject - on wall, MR-safety form - trays in clinic marked *kontrolskema*) ☐

Turn on the projector ☐

Locate button boxes (dual button-box, multi-color buttons, use ”left”) ☐

Locate tape, earplugs, & blue paper ☐

Cover stretcher paper, tear off at perforation ☐

Ensure headcoil on shelf ☐

Set up screen inside scanner ☐

Check data pie chart, if more than 3/4 delete data (only deleted data if logbook says transferred to pacs) ☐

**If first scan of the day** ☐

Arrive 30 min early ☐

Turn on scanner under quench button (~10-20min) ☐

Unlock scanner with key under quench button ☐

Turn on both computers ☐

**Greet subjects**  ☐

Remember name, use frequently ☐

Fill out kontrolskema - hold on to pen ☐

Fill out subject id form ☐

Remind entering a magnet > metal attracted into scanner ☐

Remove all metal (earrings, rings, piercings, jewellery, loose change, hairties, stuff in pockets, check turnups of trousers, condom pockets, glasses, wire in bras) ☐

Remove any unnecessary objects (even if non-metal) ☐

Double check metal removal do not take their word for it, even if experienced researcher ☐

Offer hospital gown for testing (change in locker) ☐

Place removed items in locker ☐

**Training subjects**  ☐

Remind subjects of game. Read them the instruction sheet ☐

Run ~20 trials outside of scanner or as many as necessary (’training.py’, default 20, 5mins each for passive then active) ☐

Demo what happens if they dont press *If you don’t press in time, it means the scan lasts longer* ☐

Explain time of whole experiment (3hrs total, ~60mins part 1, ~75mins part2, with breaks) ☐

**Register subject**  ☐

Fill out log book, put logno as patient id ☐

On console, set patient id to the logbook no. ☐

On console, set patient surname to subject id ☐

Project name needs to be LogUtil for PACS ☐

Load LogUtil sequence from the program card ☐

**Getting ready to enter scanner room**  ☐

Does S needs toilet? ☐

Explain S can talk anytime while the scanner is not running ☐

Explain intercom works like a walky-talky, while we talk, we cannot hear, wait before speaking ☐

Explain even small movements will impair data quality, this goes for moving body parts, esp. head & eye. ☐

Very very important to lie as still as possible ☐

Remember fixate numbers/cross at all times ☐

Don’t form any loops with your body (no crossed legs, no touching of hands) ☐

Explain possible muscle twitches when scanner runs, all normal & not dangerous ☐

Just before entry to scanner room triple check for metal items including anyone else with you (even if senior) ☐

**In scanner room - information**

Ask them to sit on bed ☐

Inform S scanner makes different noises (bleeps, knocks, grinding, buzzing, all normal) ☐

Introduce response devices (Use ”left”) ☐

Attach physiological noise (pulse & resp: make sure valve points down) ☐

Ask subject to lie down on bed ☐

Show alarm bulb, loop under resp belt, explain only if urgent ☐

Earplugs ☐

Apply padding to head (slightly tight, not too tight) ☐

Head coil, ensure eyebrows align with notch ☐

Attach mirror, make sure screen roughly centre (single mirror nearest screen) ☐

Ask them if they feel comfortable / warm enough / cushion under legs? ☐

Doublecheck everything okay before entering bore ☐

Hit autoloader ☐

Explain leaving room, and will talk on other side ☐

Check intercom & ask to squeeze alarm bulb ☐

Turn off lights in scanner room (switch 2 underneath phys noise in control room) ☐

**Data collection pre-break**

0. Start game so it waits for trigger. Do not move cursor of test computer while game is playing ☐

*1. We now run a localiser scan to localise your head, It will only be 30s, expect buzzing-grinding-knocking-bleeping noises, nothing to do just relax* ☐

2. AAhead_scout ☐

3. EPI sequence (AP phase, ~3000 volumes ~ 25min), position to hit striatum and midbrain as shown ☐

4. After shim, before final gui: *We’re now ready for your* *to play the game for 25mins it will be the same bleeping for the full duration.*☐

5. *Just relax for 2 mins, we are just going to run another localiser, again there will be various noises”* ☐

6. Reverse phase (PA) fMRI (5 volumes) [If this hits stimulation limit > calculate> adjust rise time (rather than FOV)] ☐

8. AAhead_scout ☐

9. *”We’re again ready for your* *to play the game for 25mins it will be the same bleeping for the full duration.”* ☐

10. EPI sequence (AP phase encoding, 3000 volumes, 25min) ☐

11. Reverse phase (PA) fMRI (5 volumes) ☐

12. Get S out of scanner,15min break *”Do you need the toilet, or a drink?”* ☐

13. Read instructions & demo active phase ☐

**Data collection post-break**

0. Put S back in scanner – earplugs, phys noise, alarm bulb, key press box. ☐

1. Start game ☐

*2. ”We now run a localiser scan to localise your head, It will only be 30s, expect buzzing, grinding, & knocking noises, nothing to do just relax”* ☐

3. AAhead_scout ☐

4. EPI sequence (AP phase, 3000 volumes)

5. After shim & gui: *”We’re now ready for your* *to play the game for 25mins, same bleeping noises”* ☐

6.*”Just relax for 2 mins, we are just going to run another localiser, again there will be various noises”* ☐

7. Reverse phase (PA) encoded fMRI (5 volumes)

8. AAhead_scout ☐

9.*”We’re now ready for your* *to play the game for a final 25mins, same bleeping noises”* ☐

10. EPI sequence (AP phase, 3000 volumes) ☐

11. Reverse phase (PA) fMRI (5 volumes)

*12. ”Ok nearly there, we now have a final structural scan, where you have nothing to do except lie still for 6 minutes, and then we will get you out”* ☐

13. T1 – mprage1 ☐

**Get subject out of scanner**

Move cursor on test comp to empty file ☐

Debrief subject ☐

Inform accumulated wealth for the day, and if they are coming back it will be roughly the same again ☐

**Save data**

Behavioural data (mat files & txt files, both on USB + dropbox, place in data folder with txt files) ☐

Export whole folder (fMRI, T1, phys noise) to Samba ☐

Close session on both consoles ☐

Transfer to pacs > transfer > send to > DRCMR

Check complete > transfer > network job status

Check arrived at pacs > log in

**Clean up**

Tidy everything, make it spotless, (or wake with horse-head) ☐

Place bed half way down ☐

Put coil on shelf ☐

Put new paper on bed ☐

Put all equip in right place ☐

Wipe down surfaces with wetwipes ☐

Put phys monitors on charger, check if it charges ☐

**If final subject**

Return screen to shelf ☐

Turn off projector ☐

Check log books filled out ☐

Take all paper work with you ☐

Turn off all lights ☐

Place cushions into where headcoil went ☐

Close both computers & turn off ☐

Once both computers are turned off, press ”System Off” under the quench button ☐

After pressing ”System Off”, turn the key to lock the scanner ☐

**Admin**

Put MR-safety protocol and subject code in locked draw
