# Supplementary material for: Ergodicity-breaking reveals time optimal decision making in humans
Source: PLoS Comput Biol. 2021 Sep 9;17(9):e1009217. doi: 10.1371/journal.pcbi.1009217 (PMC8454984; doi:10.1371/journal.pcbi.1009217)
Supplement: S6 Text — (DOCX) [file pcbi.1009217.s006.docx]

**S6 Text: Subject Instructions**

**Introduction.** The experiment is divided over two days, within each day there will two different phases, a passive phase and an active phase.

The main aim is to study how the brain reacts to changes in wealth. All of the money involved is real, and you will be will be paid out the total wealth accumulated, summed over the two days.

**Day 1**

**Passive phase.** For the passive phase, you will see a number in the middle of the screen, this is your current wealth for the day in kr.

When you see a white box around the number, you are to press the button within 1s. (If you do not, you will be instructed to “press button earlier”).

Shortly after pressing the button you will see an image in the background, and this will cause your wealth to change.

You are instructed to attend to any relationship between the images and the effect this has on your wealth, since in the active phase that follows you will be given the opportunity to choose images to influence your wealth.

Learning these relationships can make a large difference to your earnings in the active phase.

**Active phase**. With the money accumulated in the passive phase, you will play gambles composed of the same images.

In each trial, you will be presented with two of the images that you have learned about in the passive phase.

By pressing the buttons in the scanner to move a cursor, you now have the option to choose to either

1. Accept gamble one, in which case you will be assigned one of the two images, each with 50% probability (not shown), or…
2. Accept gamble two, in which case you will be assigned one of the two images, each with 50% probability (again not shown),.

The outcomes of your gambles will be hidden from you, and only 10 of them will be randomly chosen and applied to your current wealth.

You will be informed of your new wealth at the end of the active phase.

You can keep any money accumulated after the active phase.

If you do not choose in time, then we will give you one of the worst images, it is recommended that you always choose in time.

The decisions you make can make a big difference to your end wealth.

**Day 2**

**Introduction.** On day two, you will be endowed with a new wealth of 1000kr, and you will go through the same active and passive phases as described before, but the images will be new and they will be associated with different changes in wealth.

**At the end of the two days.** Your accumulated wealth will be added over the two days, and transferred to your account, within approximately two weeks, and is taxable under standard regulations (B-income).

Total earnings = (Wealth after day 1) + (Wealth after day 2)

This will be paid over and above your remuneration for participating in the experiment
