# Supplementary material for: Ergodicity-breaking reveals time optimal decision making in humans
Source: PLoS Comput Biol. 2021 Sep 9;17(9):e1009217. doi: 10.1371/journal.pcbi.1009217 (PMC8454984; doi:10.1371/journal.pcbi.1009217)
Supplement: Attachment — Submitted filename: ReplyToReviewersPlosCompBiol.docx [file pcbi.1009217.s007.docx]

Reviewer #1:

This paper demonstrates that (a) people’s decisions about risky monetary gambles are sensitive to the dynamic by which this money grows/shrinks (additive vs. multiplicative); and (b) this sensitive behavior closely approximates the normative benchmarks to maximize an arithmetic mean value for additive dynamics and a geometric mean value for geometric dynamics. The authors convincingly show that a model whose utility matches this normative benchmark depending on the dynamic vastly outperforms more standard utility models that do not consider dynamics, such as prospect theory.

I have been incredibly impressed by this manuscript since it was released as a preprint a year or so ago, and this is one of the rare cases where I do not have substantive comments about the paper’s quality. Even without the complex Bayesian modeling, the paper makes a very compelling case that multiplicative dynamics dramatically change people’s risk preferences in an optimal way — a point that seems to have been largely ignored in the decision-making literature that I am aware of. (Perhaps this has been studied in some detail in the financial literature, as relates to the Kelly criterion, but this is not my expertise.)

**We thank the reviewer for their enthusiastic evaluation.**

My only (minor) worry is about the paper’s comprehensibility. Specifically, the figures are quite detailed and often include a lot of detail about the specifics of the Bayesian model fits that were used. Although this is useful content to include for people who are familiar with this style of modeling, it seems like some of it would be more appropriate for the supplement.

**We agree some of the material is better suited to the supplemental. That said, there are some specific plots we would like to argue for keeping, which we detail immediately below.**

For example, many of the robustness checks in Fig. 2 seem like they would fit better in the supplement in order to put more emphasis on the really critical subpanels like (a).

**We have a preference that the robustness checks should be foregrounded for key statistical tests and that placing them in the main text communicates the importance of them. The results we present are controversial in some fields and we need to demonstrate the carefulness with which we have performed the analyses. Indeed, some of our discussions with other reviewers have hinged around these plots, thus reinforcing their centrality.**

**A common criticism of the Bayesian statistics we have used is their dependence on the priors. The third column of panels Fig. 2 shows the reader that the strong evidence observed is ~invariant over a large range of prior widths. Showing this in the main text communicates right from the start that sensitivity to prior widths is not a valid criticism.**

**Similarly, another common criticism of our experiment is that it is underpowered. The sequential analyses in the fourth column of panels shows that strong evidence is exceeded after around half of our small sample. Furthermore, given that in the behavioral sciences there is widespread concern about replication and the robustness of statistical practices, we argue that foregrounding such analyses in the main text is important for contributing to future norms.**

Similarly, Fig. 3 requires a huge amount of effort to process, and I’m not so sure panels like (i) and (j), which look at order effects, need to be in the main text, given that order did not seem to play a major role on behavior.

**Yes, we agree this helps emphasise the more important results. We have now put 3h-j into the supplemental (now Supp. Fig 5). In the spirit of this suggestion we also move panels 3l-n (Supp. Fig. 6)**

This would help put emphasis on the critical subplots like (c), which show how much better the time-optimal model performs relative to a model with static risk aversion.

**We hope that you find that removing these 6 panels from Fig. 3 helps emphasise the more important subplots.**

Critically, I believe the main findings of this paper should be comprehensible to people who don’t have a strong background in Bayesian modeling. Even without the Bayesian analyses, it should be quite clear from the figures that dynamics matter in the predicted ways.

Reviewer #2:

The paper presents a behavioral experiment, where subjects first passively learn values of fractals (which could be additively or multiplicatively increasing their endowment – representing the two main conditions of the experiment) and then make forced binary choices between 50/50 gambles that include those fractals. At the end of the active phase, 10 gambles are selected at random and applied to their initial endowment.

The paper argues that when dealing with multiplicative gambles, individuals tend to use log utility, while when dealing with additive increases, they behave as if they employ linear utility. These results are supported by a series of Bayesian statistical analyses and model fits using various utility models.

While I do not question the results of the experiment in general (although see power considerations below),

**We deal with power below.**

and I think it’s carefully implemented (and the model fitting exercise is impressive),

**Thank you.**

I unfortunately (1) do not believe it is sufficiently novel to justify publication in PLOS Computational Biology;

**Our paper is novel. To our knowledge this is:**

1. **The first experiment to explicitly test the effect of gamble dynamics on risky decision making**
2. **The first demonstration of risk preferences changing as function of changing dynamics**
3. **The first ever experimental test of ergodicity economics, itself a nascent branch of economics**
4. **The first ever use of dynamical conditioning, in which dynamics are learnt implicitly prior to risky decision making**

**Other elements of methodological novelty include:**

1. **The large incentives offered to subjects - we know of no other experiment in experimental economics offering higher than a maximum winning of $600.**
2. **The number of decisions made, exceeding 600 per subject which is extreme for experimental economics.**
3. **The use of hierarchical latent mixture models to directly evaluate the evidence supporting decision models of differing complexity.**

**If novelty is an important criteria to PloS CB, then the above is an argument for the novelty of our paper.**

(2) do not see how the results of this specific experiment contribute to our understanding of economic behavior in dynamic settings. I wish I could be more positive, but I think the situation would be different if the neuroimaging results were presented as well.

**The results contribute to our understanding of economic behavior by showing that ergodicity has a large and systematic influence on risky decision making. This shows that a simple null model of maximising time average growth explains choices better than existing descriptive models. The insight this offers is that, to a first approximation, people preferring their wealth to grow at a faster rate is a better explanation of risky decision making than the mainstream models which are more complex, and yet offer no principled explanation for why utility functions look the way they do.**

**This is a basic but important contribution to our understanding of economic behavior.**

- First and foremost, the novelty of the *ergodicity* considerations (as well as the works of Ole Peters) is strongly disputed by economists. Maximizing the expected value of the logarithm of wealth is known in the finance literature since 1950s as the Kelly criterion and has been studied in numerous publications on portfolio optimization (note though that the use of log utility does not guarantee success in all cases, and the expected utility theory still can be a viable alternative). The macroeconomic and asset pricing literatures both use log utility in a wide range of models.

**Our main result is not simply that log utility is a good fit under multiplicative dynamics. Our main result is that risk preferences change from ~log utility to ~linear utility as predicted if agents are maximising time average growth.**

**This was a novel theoretical prediction, published in 2016, before this experiment was conducted. It is a prediction not made anywhere else in the economic literature, and it is incompatible with any class of theory which assumes stability of preferences. In other words the dependency between dynamics and utility contradicts a foundational assumption made by nearly all, if not all, utility theories.**

**One of the other novelties of the ergodic decision theory approach is that it generalises the Kelly criterion to any dynamical setting. It thus deepens our understanding of the Kelly criterion by generalising it to a much larger class of optimisation behaviors, only two of which we test here.**

The standard expected utility theory does not require ergodicity and has little to do with it. That being said, the laboratory evidence on dynamic gambling behavior and log utility is quite limited (for a rather weak example, see Haghani and Dewey (2016), Rational Decision-Making under Uncertainty: Observed Betting Patterns on a Biased Coin), so this might be a potentially fruitful line research. However:

- I cannot see how the dynamics are really represented in the experimental design of this study. In the passive task, the dynamics are quite clear: subjects observe their wealth change in real time, going up and down, in order to learn the values associated with the fractals. In the active task however, there is no real dynamics – as far as I understand.

**The dynamics exist in the active task insofar as the fractals impose dynamical changes in wealth when they are realised at the end of the experiment.**

The subjects make choices between gambles without any feedback, so they pretty much make these choices completely independently. At the end of the task, 10 random choices are selected (this number seems important, but is never justified), gambles are realized, and the results are applied to their initial wealth level, all at the same time (sequentially, in random order? - again, if I understand the methods correctly).

**The order of the realisation does not matter, since it is of the form:**

**wealth + a + b +... n**

**or**

**wealth * a * b *... n**

**Permuting the order of (a & b &.. n) yields the same sum (or product).**

**When realising at the end of the experiment, it makes no difference whether they are applied all at once e.g. wealth + (a + b +... n), or sequentially (wealth +a, then that new wealth +b…), since you get the same answer. The same holds for multiplication.**

How does this correspond to any dynamic optimality considerations as presented in Figure 1f-g (also see a minor point below – does the optimality effect really require the time horizon of weeks and wealth of millions of crowns?

**Great question. It is surprising it has never been asked before, especially given our emphasis on optimality.**

**The optimal strategy is as optimal for a week as it is optimal for a single decision. If you look closely at the plots 1f-g, you will see that the green line is the optimal path coming all the way back to the origin so it is clear that this is true at the scale of hours. We also show data that the closer to time optimal the strategy, the higher the time average growth rate realised within the experiment (detailed immediately below).**

Does it mean that the real effect of using log utility in the task is economically insignificant?)?

**The actual monetary effects on take home pay depend on the wealth level at the end of the passive phase, which was randomised. It is therefore the time average growth rate that is the most meaningful statistic to compare between subjects.**

**The effect of the risk aversion parameter on the growth of wealth at the timescale of the experiment is visible in figure 2l (now Supp. Fig. 6a), where it can be seen that the closer the subject is to time optimal value of risk aversion, the highest time average growth rate is achieved. (Note that this is not circular because we are comparing a theoretical optimum, with an achieved time average growth rate, which is a random variable on this timescale).**

**In Fig. 2m (now Supp. Fig. 6b) and Fig. 2n (now Supp. Fig. 6c) an increasing deviation from the time optimal value of risk aversion decreases the time average growth rate achieved in the game. Both correlations were negative and obtained Bayes factors ~10 and ~30 respectively for additive and multiplicative dynamics. These are economically meaningful differences in growth rates on the timescale of the game itself.**

It seems that when subjects are making a choice in each specific trial, they do not know the initial wealth level these choices would be applied to

**Subjects know their initial wealth when they start the active phase. They are choosing such that their choices have a chance of being realised at the end, as per our instructions.**

(since, I assume, all 10 choices are applied sequentially at the end, with the wealth endowment as the initial value)?

**As mentioned above, the 10 choices are realised at the end, but it makes no difference whether they are applied all at once at the end (wealth + (a + b +...), or sequentially at the end (wealth +a, then that new wealth +b…), since you get the same answer.**

**Note that it is not the endowment that is the initial value of wealth for the active phase, but rather how much they had at the end of the passive phase, which was a random value within bounds.**

To summarize the argument, the task design does not seem to capture the idea it is supposedly aimed to demonstrate: that people, when making decisions in a *multi-period* environment with gambles applied multiplicatively to their wealth, do that optimally (using log utilities, or the Kelly criterion). Yes, the experiment shows that log utility functions fit better in the multiplicative case. However, it is not at all clear that log utility is optimal specifically for this experimental setup (this needs to be clearly shown for the specific gambles used in the task, and all possible initial wealth levels), and how these results generalize to a truly dynamic setting.

**The simulation in Fig. 1f & g is for the setup used in this experiment. It takes a range of synthetic agents with different risk aversion parameters and shows how their wealth fares playing this experiment, when it is continuously repeated back to back. These repetitions of the experiment are not identical because the order of gambles is stochastic, as it is in the real experimental game.**

**Log utility allows one to compute the time average growth rate for multiplicative dynamics, and thus maximising it in this game gives the log-utility-agent the highest time average growth rate. Likewise for linear utility and additive dynamics. The fact that the superiority of the time optimal agents growth rates persist over a large range of wealths within this simulation does indeed demonstrate that this holds for all possible wealth levels, though to prove this one needs to resort to the mathematical arguments presented in Peters & Gellman 2016.**

The study is clearly underpowered. The main result is the difference between a choice proportion of 0.49 (for the additive case) and a choice proportion of 0.71 (for the multiplicative case), with SD being around 0.2 in both cases. I understand and appreciate the Bayesian exercise, as well as the model fits, but in the end, everything just comes down to these two proportions.

**The proportion result is not the main result, it is one of three main analyses. The parameter estimation and model comparison (Latent mixture model) being the other two. The latter two are much stronger tests of the theoretical disagreement between ergodic theory and mainstream utility theories.**

**The proportion result does of course have the advantage of being “model independent”, however demonstrating a difference in proportions is only compatible with time optimality, it does not test the quantitative predictions of time optimality, and it does not compare the predictive adequacy of the competing theories.**

Power concerns are only mentioned once in the Methods section: “The sample number was based on general guidelines for the minimal number of subjects required for medium effect sizes in neuroimaging datasets”.

**This is discussed in the discussion section “statistical considerations”. Power is mentioned where we discuss our experimental design requirement to balance group size and within subject repetitions, due to the nuanced nature of the model comparison (and also the budget constraints due to high payouts).**

And if one does the power calculations for these numbers, they do not look great.

**Power is an important concept when experimental planning, since on average, higher powered experiments yield more informative results than lower powered experiments. Indeed, this is why we are in the process of replicating this experiment, by expanding power (more accurately, the chances of obtaining strong evidence) both within subjects and across subjects for more demanding inferences.**

**However, it is not valid to use power to critique the evidential support offered by data once the data are observed. (Full disclosure: Many of our author team confess to having made this same critique)**

**This is nicely described by Wagenmakers et al. (2014, Behavior Research Methods) in their paper “Power fallacy”. We quote from the conclusion which makes our point:**

***“With the data in hand, statistical inference should use all of the available information, by conditioning on the specific observations that have been obtained (Berger & Wolpert 1988; Pratt 1965). Power is best understood as a pre-experimental concept that averages over all possible outcomes of an experiment, and its value is largely determined by outcomes that will not be observed when the experiment is run. Thus, when the actual data are available, a power calculation is no longer conditioned on what is known, no longer corresponds to a valid inference, and may now be misleading.***

***Consequently, as we have demonstrated here, it is entirely possible for low-power experiments to yield strong evidence, and for high-power experiments to yield weak evidence. To assess the extent to which a particular data set provides evidence for or against the null hypothesis, we recommend that researchers use likelihood ratios or Bayes factors. After a likelihood ratio or Bayes factor has been presented, the demand of an editor, the suggestion of a reviewer, or the desire of an author to interpret the relevant statistical evidence with the help of power is superfluous at best and misleading at worst.”***

**If you are concerned about the level of uncertainty in terms of the predictive adequacy of the statistical models in question, then this is contained in the Bayes factors we report.**

**So what do we observe in our experiment? We plot all our Bayes factors sequentially, as the subject numbers increase. For the proportion results in Fig. 2g & 2k, we see that strong evidence is exceeded after only ~half of our small sample. The full sample yields very strong evidence. The Bayes factor contains what we need to assess the relative predictive adequacy of the competing models, and in our case this is very strong evidence for the statistical comparisons that are being questioned.**

But this is not a neuroimaging study, this is a behavioral study. Being honest, I do not really understand why this is being published separately from the neuroimaging results since it would be a stronger and more interesting paper if those were combined. In 2020, publishing a behavioral effect based on 18 subjects with 320 trials each

**Correction: 624 trials per subject, which is a higher number of choices per subject than we were able to find for any other experiment inferring risk preferences. Furthermore each subject faced 320 unique gamble choices, again more than any other equivalent experiment we could find. (Exceptions include animal studies where tens of thousands of choices may be obtained). Finally, these gambles spanned a large range of possible monetary outcomes, again maximising the chance of a large effect size. Again this was a much larger range of losses and gains than is typically ever tested in the relevant fields.**

(with only 25 trials actually measuring the effect)

**All 624 trials per subject are used in the parameter estimation and model comparison, and thus are important for measuring the effects of interest. Simulations performed when optimising our experimental design indicated that a smaller gamble space and fewer trials would not be sufficiently discriminatory between models.**

is not really up to the current standards of replicability (even though the analyses were preregistered).

**The fact that there are only 18 subjects is why we are careful not to extend our inferences to a wider population, and that we restrict our inferences to the subjects tested. The standards of replicability are not solely assessed in terms of the number of subjects, but on balanced consideration of effect size, subject numbers, and within-subject repetitions.**

**There are subfields in which this imbalance is more extreme such as psychophysics, and single cell neurophysiology, where large volumes of data is collected from 2-3 animals, or 3-6 subjects. Notably these subfields have been less vulnerable to replication crises.**

**We thus defend our choice to concentrate more of our data collection within subjects, given our inferential needs.**

Minor remarks:

- Prospect theory *is not* a utility model; it’s not a well-defined utility function, it’s a value function. This mix-up happens throughout the manuscript.

**This was a deliberate choice. We note this in the modelling section where we introduce Prospect theory.**

***“Note that, although this is referred to as value within prospect theory itself, we here refer to this as utility for clarity of comparison between models.”***

**To expand on this. Whilst it is true that the authors of Prospect theory chose to name this function a value function, there are other reasons it is appropriate to refer to it as a utility function. First, it plays the role of a utility function in identical ways to the other two theories we compare, and thus for conceptual clarity we call it a utility function. Second, calling it a utility function fits with at least some existing conventions, in which value functions are reserved for certain outcomes, and utility functions for outcomes with risk.**

- I am not sure if Figure 1e has any value since the reader does not know what these fractals correspond to.

**Fig. 1e is meant to illustrate that discrepant trials are just like the other trial indicated in 1a, but that with the added detail of them having this pattern of preferences for different utility functions. In this sense, the reader doesn’t really need to know what the fractals mean.**

- Figure 1f-g is not clear: the colors are difficult to tell apart, a legend could be useful.

**Thank you, yes we have now added a legend and arrows to make this clearer.**

For figure 1g – do I understand correctly that the effect is only clearly visible for wealth levels of 10^100 crowns?

**We address this same point earlier in our response. In short, the optimality of the utility function traces all the way back to the origin, and is visibly optimal after a few hours. Further, we show in further analyses (again detailed above) that the optimality effect is apparent on the timescale of the experiment.**

That doesn’t make much empirical sense. How do these calculations look like when they are applied to the stakes used in the experiment?

**These calculations are applied to the stakes used in this experiment.**

- End of the introduction: “impose a consistent effect on utility functions” – would be better to specify here what the effect is and why it is assumed that the utility functions are causally affected by the experimental manipulation.

**Thanks. We have changed the introduction to state the following:**

**“We found convergent evidence that gamble dynamics impose a consistent effect on utility functions, with participants shifting from approximately linear utility functions in the additive session, to approximately logarithmic utility functions in the multiplicative session. The pattern of these effects are better approximated by a time optimal model compared to standard utility models.”**

**We prefer to leave the inference about causality and possible confounds to the discussion, as the end of the introduction is meant as a brief summary, rather than a defense of the inferential logic.**

Reviewer #3:

Thank you for your comments.

In this paper, the authors present a model-driven analysis of decision making under risk in two types of scenarios: additive outcomes where wealth is increased or decreased by a particular amount, or multiplicative outcomes where wealth is increased or decreased by a particular proportion. The authors show – through choice proportions, parameter estimates, and formal model comparisons via model indicator variable – that participants adjust their decision strategies across these two conditions, corresponding to a time-optimal utility model.

In general, I found the methods and analyses thorough. The Bayesian analyses were carried out in a rigorous way by checking robustness to different priors, reporting both the Bayes factors and credibility intervals, and performing comparisons on both the raw data and the models. Unusually, I didn’t really see any missing analyses or conceptual flaws in the paper, so most of my comments will center on clarification and style rather than pertain to the substantive conclusions.

I found the results and discussion convincing, but I think the exposition in the introduction could be more comprehensive.

I struggled to understand from the single example how time average growth rate is calculated, and why a logarithmic utility function is time optimal. Going through the calculations and the predictions of different utility functions as they relate to dynamic changes in wealth would be helpful.

**Yes, we are happy you mention this. It lost some comprehensiveness due to word restrictions in previous submissions. Fortunately, PLoS CB allows any length as long as it is concise. We thus add the following changes to the introduction:**

**We hope that this provides a fuller explanation.**

Another change that might help clarify the content of the paper is if the model descriptions were provided in the introduction, as opposed to materials & methods. To an extent, the order of the sections for the journal is working against the authors here – my interpretation of the ordering is that materials & methods should be details needed to replicate exactly the experiment and results in the paper, but not details that are necessarily critical to understanding the results themselves. The authors may have to work around this by re-arranging the paper to an extent. Reading linearly through the paper, I had no idea what delta-u meant, or where nu was included in the models, or exactly what the distinction between isoelastic utility and time optimal utility models were. These are of course all provided in the materials & methods section, but it would be better suited as part of the intro – if only to avoid having to constantly flip back and forth between the results and methods. In addition to being critical to understanding the results, setting up the model predictions in the intro should also help motivate the experimental and analysis approaches (particularly the additive-multiplicative conflict trials), helping the reader understand why the findings are interesting.

**Yes this is a nice suggestion, we also would prefer the methods and models to come first. We are allowed to if we have good reason, so we will use your arguments, as well as our own, to request this with the editor. We here submit the manuscript with this ordering, though we accepted the changes so that other more important changes are still visible.**
