# Supplementary material for: Ergodicity-breaking reveals time optimal decision making in humans
Source: PLoS Comput Biol. 2021 Sep 9;17(9):e1009217. doi: 10.1371/journal.pcbi.1009217 (PMC8454984; doi:10.1371/journal.pcbi.1009217)
Supplement: Attachment — Submitted filename: ergChoice_ReviewerResponse_Resubmission2.docx [file pcbi.1009217.s008.docx]

**Response to the Editor, 2nd revision**

**In our last resubmission, we had satisfied reviewers 1 and 3, and we received comments only from reviewer 2.**

**Reviewer 2 felt that we had not substantively changed the paper in accordance with their comments, and further that a new reply had recently come out (Doctor, Wakker & Wang 2020), outlining criticisms of the experiment that should be addressed.**

**In our 2nd reply here, we go back through all of the reviewer 2’s original comments, and we substantively update the paper accordingly.**

**In addition, we go through all of the points made by Doctor et. al. replying in full in the supplementary discussion.**

**Reviewer #2 - 2nd round of review:**

Interesting that the authors chose to "talk" themselves out in response to virtually all of my comments instead of making (sometimes perhaps just small and/or clarifying) changes to the paper, even in the cases where they are objectively wrong (such as calling the prospect theory a utility function).

**We appreciate we should have made greater efforts to change the paper in line with your questions and comments. We apologise for any frustration caused. Going back over your previous review we have made the following additional changes to the paper:**

**In response to your question about novelty we added the following section:**

**In response to your concerns about logarithmic utility already being widely used, thus attenuating the novelty of the results we have added our response to this into the discussion:**

**In response to your question about where are the real dynamics in the active task, we now write this in the methods summary:**

**In response to your comment about not justifying the number of realisations, and also your question about the order of realisation, we now write this in the section “subject payout”:**

**In response to your question about whether the optimality depends on the time horizon, we now write this in the figure legend for 1f and 1g:**

**In response to your question about whether the differences in risk aversion are economically meaningful on this time scale we have edited the appropriate section discussing this:**

**We think this is an important point so we also promote it to the main discussion section on “Logarithmic utility”:**

**In response to your question about power we now write in the discussion:**

Just a few days after I submitted my initial review, Nature Physics published a response to Ole Peters' paper, written by several economists:

Doctor, Jason N., Peter P. Wakker, and Tong V. Wang. "Economists’ views on the ergodicity problem." Nature Physics 16.12 (2020): 1168-1168.

**Yes, we provided detailed comments on this reply and advocated for its publication. We had planned to reply in a separate paper, but we are happy to do so here. We list our responses and changes to the paper at the end of this response.**

This paper also provides a series of very strong criticisms for the paper we are discussing now (see Appendix A in the Supplements), and I must suggest that the authors should address these before th e paper is published (although I understand it might be impossible with the current design). Some of them mirror my comments (perhaps in a more convincing and clear way).

To be more specific, when the authors respond "When realising at the end of the experiment, it makes no difference whether they are applied all at once e.g. wealth + (a + b +... n), or sequentially (wealth +a, then that new wealth +b...), since you get the same answer.", this is simply not true.

**I can’t imagine that we are disagreeing about arithmetic, so I assume there is some other source of misunderstanding between us. I will try my best to explain better. Illustrated for two outcomes rather than 10:**

**W1 = W0 + a;**

**W2 = W1 + b; (sequentially)**

**= W0 + a + b (all at once)**

**In the above, the two ways of obtaining W2 are identical in the additive condition.**

**W1 = W0 * a**

**W2 = W1 * b (sequentially)**

**= W0 * a * b (all at once)**

**In the above, the two ways of obtaining W2 are identical for the multiplicative condition.**

The order *does* matter if the outcomes are realized sequentially, and this is the whole point of investigating dynamic decision making.

**The order does not matter to the final wealth in this particular setting.**

**W2 = W0 * a * b	(a before b)**

**= W0 * b * a	(b before a)**

**The same is true for the additive condition.**

**It would matter to decision makers if the outcomes were realised during the game, and they were revealed to the subject at the time of their occurence, because this would update their knowledge of their current wealth. Clearly changing the order changes the sequence of wealths within the game, which could plausibly change the pattern of decisions made.**

**Maybe that is the crux of our disagrement?**

**In any case, we now include discussion of this point to hopefully make this clearer to the reader:**

The Doctor et al. response raises the same concern; this is the single major flaw of the design.

**We discuss this response in a new section in the supplementary discussion section entitled “Reply to Doctor, Wakker and Wang”, p14 in the supplementary information file.**

Finally, if the claimed novelty includes "The first ever experimental test of ergodicity economics, itself a nascent branch of economics", I would strongly suggest to submit the paper to an economics journal, if the authors still wish to receive feedback from a qualified audience most familiar with the subject.

**We would like to communicate broadly with economists, psychologists, physicists, mathematicians, and computational biologists. Plos CB has the advantage that some of its readers and reviewers are familiar with both decision theory, cognitive models, and with hierarchical Bayesian methods. If the journal is happy with our paper, we are enthusiastic to engage with their audience.**
