# Supplementary material for: Ergodicity-breaking reveals time optimal decision making in humans
Source: PLoS Comput Biol. 2021 Sep 9;17(9):e1009217. doi: 10.1371/journal.pcbi.1009217 (PMC8454984; doi:10.1371/journal.pcbi.1009217)
Supplement: Attachment — Submitted filename: ergChoice_ReviewerResponse_Resubmission3.docx [file pcbi.1009217.s009.docx]

**Response to the Editor, 3rd revision**

**In the last round, Reviewer 2 made some important points about the external validity of the experiment.**

**We reply to these comments and have acknowledged these points in the revised manuscript. We have not made any changes to the supplementary information since the last revision.**

**In addition to changes made in response to reviewer 2, we discovered an error in equation 11. This has no consequences for any of the results since it was only an error in the presentation of the formalism.**

**We now introduce this equation more fully in the following section:**

**Note that eq 11 has changed but is not highlight due to a bug in MS word.**

**Reviewer #2 – 3rd round of review:**

Reviewer #2: I appreciate that the authors made the effort to edit the manuscript in response to my previous comments. I have read their new response as well as the response to Doctor et al.

My new comment will be more of a methodological discussion rather than a direct comment on the details of the paper. I do completely understand the experiment that was run, and I do not really have any issues with the procedure. I would say my issue with the paper lays primarily in the interpretation of the results and implied importance of the findings. I find it difficult to provide a specific recommendation, but I hope that my thoughts could be useful for the editorial decision – and could help to understand why there is such a backlash against this line of research among economists.

**We thank the reviewer for these careful comments.**

There is a reason economists dislike laboratory experiments (if we do not count those who run lab experiments themselves). In many cases, the findings have very little external validity. It is a tired criticism, but it is sometimes still appropriate. Specifically, it is very difficult to run a macroeconomic experiment, or an experiment that deals with individual “wealth”. Real decisions over wealth take days, months, and sometimes years. The stakes are extremely high for the individual that makes these decisions (it’s their wealth after all). These stakes are often present in macroeconomic models (e.g. see Epstein-Zin preferences), but very few try to test them in the lab – and if they do, they provide very little actual value to the community. The reasons are obvious: the “wealth” in the lab is not actual wealth, it’s pennies; the decisions do not matter for the subject’s economic well-being in the long run; the decisions are not made with the same level of carefulness as the real-life decisions.

When we test a simple risk choice task in the lab, there are many real-life decisions that could correspond to the task; even buying a lottery ticket. When the paper claims that subjects dynamically estimate their “wealth” trajectory, that terminology is just not appropriate.

**Absolutely. We agree it is important to distinguish in-game from out of game wealth. We now call it in-game wealth at first mention (and indicate wealth hence) and then reminding the reader that it is in-game wealth in the discussion. The abstract now reads:**

**We repeat this at the start of the methods section:**

**And we remind the reader of this in the discussion as follows:**

There are no “wealth” decisions to be made, the subjects simply do a series of extremely fast choices about some pocket money… without even knowing their current “wealth” (i.e. endowment) level.

**We could not agree more about the problem of pocket money, or “peanuts” as we call it in the paper. This is the reason we went to the effort to ensure it was not pocket money. The money subjects can obtain is a substantial portion of their monthly expendable income and is far more than their possible earnings in one day.**

From the supplements:
“This is because the subjects did not know the following five details necessary to perform a backward induction from terminal wealths: a) how many gambles they would face; b) what gambles they will face; c) the outcome of each gamble; d) which choices would be realised; e) what their current wealth is at the time of choosing.”

So the subjects did not even know their current “wealth” at the time of choosing. I still fail to see how this reflects a “dynamical” decision-making process. It seems that the subjects were acting on extremely limited information; something that they would not do in real life, when making choices that determine their future wealth.

From the response:
“It would matter to decision makers if the outcomes were realised during the game, and they were revealed to the subject at the time of their occurence, because this would update their knowledge of their current wealth. Clearly changing the order changes the sequence of wealths within the game, which could plausibly change the pattern of decisions made. Maybe that is the crux of our disagrement?”

I see that the authors realize themselves that that observing individual outcomes could be important to the subjects – as it is to decision-makers in real life. I can hardly imagine a scenario where this information would not be available to a household. I can’t see the reason why this was not made an explicit feature of the experimental design. But again, an ideal experiment that would test the ergodicity theory would use much higher sums of money (approaching the monthly income of the subjects);

**We already did this. The possible earnings were approaching the monthly disposable income of the students. Participants can walk away with up to 750 USD. This is a substantial part of a Danish university student’s monthly budget (nearly all participants were university students). While not being a full-time monthly salary, it exceeds part-time monthly salaries. The incentives at stake were substantially higher than any other experiment on risky decision making that we have been able to find, at least in western contexts.**

longer time horizons; choices that do not take seconds, but at least hours

**(Incidentally, we now have funding to test this paradigm with extreme incentives over days and weeks, so yes, we totally agree this is an important direction.)**

I hope that my point is clear. I do not think that this experiment tests exactly what the authors are hoping it should test (decisions over wealth);

**We are not hoping it tests decisions over wealth. We infer that time optimal decision making seems evident for in-game wealth decisions that are substantial relative to their real wealth. The fact that this is possible for in-game wealth speaks to the plausibility it could apply to real. wealth decision making, but our experiment does not test that. We capture this in the following:**

**Thanks to your suggestion we now state in the abstract, the first paragraph of the methods, as well as the discussion, that we are talking about in-game wealth. We now also state that the in-game wealth may exceed their monthly disposable income. The discussion section now reads:**

it opens a potentially interesting topic, but it is unlikely make an impact for the economic literature due to these methodological concerns.

**In-game wealth and the problem of peanuts is a problem for virtually all experimental economics experiments. At the very least, by having such high incentives, we have minimized this problem, relative to prior efforts in the field.**

I think it would be beneficial for the manuscript to discuss the limitations of the experiment (and its relation to real-life decisions or external validity) in greater detail,

**Yes. We now extend the limitation section as follows:**

**and**

but ultimately it is up to the editor to decide whether the paper makes a valuable contribution. My impression is that unfortunately the experiment itself (as it was done) does very little to increase our knowledge regarding how dynamic economic decisions are made. However, it might generate an interesting discussion in the literature – so there might be some value in that!
